# Circulating fatty acids, genetic susceptibility and hypertension: a prospective cohort study

**DOI:** 10.3389/fnut.2024.1454364

**Published:** 2024-10-31

**Authors:** Lingling Lu, Xiaoli Gu, Daheng Yang, Bingjian Wang, Guangfeng Long

**Affiliations:** ^1^Department of Infectious Disease, Children’s Hospital of Nanjing Medical University, Nanjing, China; ^2^Department of Party and Government Office, Children’s Hospital of Nanjing Medical University, Nanjing, China; ^3^Department of Clinical Laboratory, Children’s Hospital of Nanjing Medical University, Nanjing, China; ^4^Department of Cardiology, Huai’an First People’s Hospital Affiliated with Nanjing Medical University, Huai’an, China

**Keywords:** plasma fatty acids, hypertension, polygenic risk score, additive interaction, cohort study

## Abstract

**Background:**

Combining genetic risk factors and plasma fatty acids (FAs) can be used as an effective method of precision medicine to prevent hypertension risk.

**Methods:**

A total of 195,250 participants in the UK Biobank cohort were included in this study from 2006–2010. Polygenic risk scores (PRSs) were calculated for hypertension using single-nucleotide polymorphisms (SNPs). Concentrations of plasma FAs, including polyunsaturated fatty acids (PUFAs), monounsaturated fatty acids (MUFAs) and saturated fatty acids (SFAs), were tested by nuclear magnetic resonance. The Cox model was used to test for the main effects of PRS, different plasma FAs and their joint effects on hypertension. Relative excess risk due to interaction (RERI) and the attributable proportion due to interaction (AP) were used to test the additive interaction.

**Results:**

Plasma PUFAs, n-3 PUFAs, MUFAs and SFAs were related to the risk of hypertension (PUFAs: HR, 0.878; 95% CI, 0.868–0.888; MUFAs: HR, 1.13; 95% CI, 1.123–1.150; SFAs: HR, 1.086; 95% CI, 1.074–1.098; n-3 PUFAs: HR, 0.984; 95% CI, 0.973–0.995). Moreover, an additive interaction was found between PRS and plasma FAs, which could contribute to an approximately 10–18% risk of hypertension, and the associations between high plasma MUFAs and a high PRS of hypertension were the strongest positive [RERI: 0.178 (95% CI: 0.062, 0.294), AP: 0.079 (95% CI: 0.027, 0.130)].

**Conclusion:**

Increased plasma MUFAs or SFAs and decreased plasma PUFAs or n-3 PUFAs were associated with hypertension risk, especially among people at high genetic risk.

## Introduction

Hypertension is the single contributing factor for the incidence rate and mortality of cardiovascular disease (CVD), including coronary artery disease (CAD), stroke, heart failure (HF), atrial fibrillation (AF), chronic kidney disease (CKD) and end-stage renal disease (ESRD), worldwide (1, 2), and more than one billion individuals are afflicted by hypertension. By 2025, the proportion of individuals with hypertension in the global adult population will reach approximately 29% (3). SBP is the main risk factor when it is ranked by disability-adjusted life years (DALYs) attributable to risk globally, resulting in 10.4 million (95% CI: 9.39–11.5) deaths and 2.18 million (95% CI: 1.98–2.37) DALYs (3).

Recently, the Lancet Hypertension Committee proposed that a healthy environment and healthy lifestyle can effectively prevent hypertension (4). Recommendations of blood pressure guidelines, proposed by the American College of Cardiology (ACC)/American Heart Association (AHA) and the European Society of Cardiology (ESC)/European Society of Hypertension (ESH), mention that changing unhealthy lifestyles can be an effective measure to prevent hypertension, including healthy diets (5, 6). Prevailing dietary guidelines, such as the Dietary Approaches to Stop Hypertension (DASH) and Mediterranean diets, emphasize reducing total fat and saturated fatty acids (SFAs) and increasing fish and olive oil with monounsaturated fatty acids (MUFAs) and polyunsaturated fatty acids (PUFAs) (7–11). However, evidence on the associations of circulating fatty acid (FA) with hypertension risk is inconclusive and insufficient (12–15).

On the basis of a previous study, the inconclusive and inconsistent relationship between dietary FAs and hypertension may be explained by gene-nutrient interactions and gene polymorphisms (16–18), and individuals’ genetic makeup may constitute a varied relationship between dietary FAs and hypertension. Large-scale genome-wide association studies (GWASs) have revealed that genetic polymorphisms also play a significant role in the development of hypertension (19–21) and have identified some genomic loci associated with hypertension, in which single nucleotide polymorphisms (SNPs) are aggregated into polygenetic risk scores (PRSs) that can sharply discriminate hypertension risk (21–24). However, because of the distinction between dietary FAs and plasma FAs, little is known about whether genetic variants could modify the specific role of plasma FAs in hypertension development.

Using large-scale sample data from the UK Biobank, to fill this gap, we conducted a prospective cohort study to reveal the relationship between plasma FA levels and hypertension. Furthermore, to evaluate the interaction between plasma FAs and genetic predisposition to hypertension, we calculated the PRS of hypertension and explored the relative excess risk due to interaction (RERI) and the attributable proportion due to interaction (AP) between plasma FAs and PRS. To our knowledge, the present study is the first to discuss the relationship between plasma FA levels and genetic predispositions.

## Patients and methods

### Study design and population

**Figure 1:**
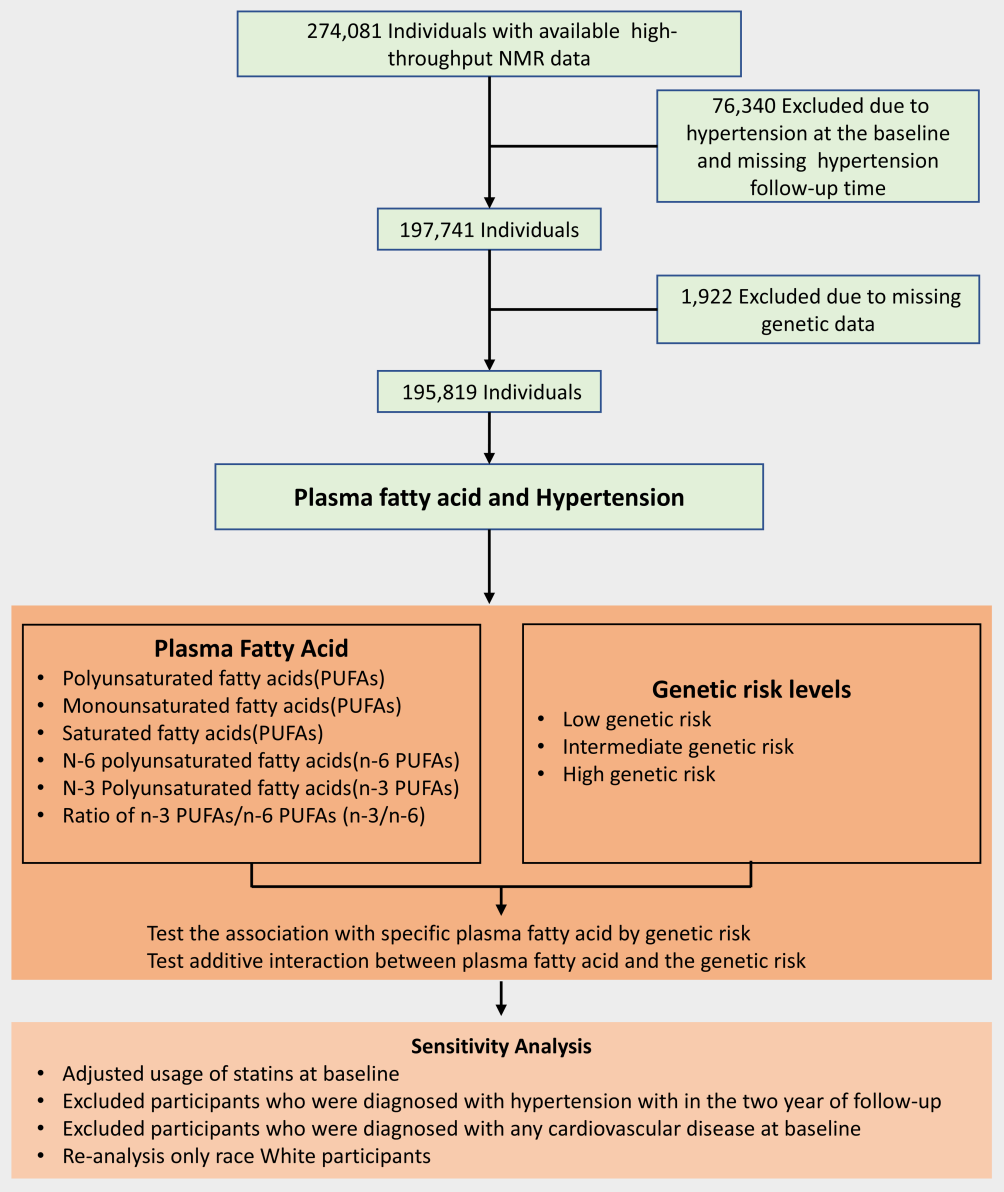
Flow diagram for the inclusion of participants.

The UK Biobank was a prospective cohort study whose research design and population details were as described above (25). A total of approximately 500,000 adults aged 40–69 years were included through multiple assessment centers from 2006–2010 (26). At baseline, participants provided electronic signatures, completed a touch-screen questionnaire, were asked about their medical history and health status by trained professionals and further collected data after a follow-up interval of 6 months to 3 years (25, 27). A flow chart of the details of participant inclusion is shown in Figure 1.

### Assessment of plasma fatty acids

The detailed scheme for sample collection and quantification of the metabolic biomarker profiling platform on the basis of high-throughput nuclear magnetic resonance (NMR) was described in a previous study (28–30) and is available at https://biobank.ndph.ox.ac.uk/showcase/label.cgi?id=220. The plasma FAs were among the 249 metabolic biomarkers. The concentrations of plasma FAs were measured repeatedly in approximately 155,000 participants and were moderately correlated with the concentrations measured at baseline, indicating the stability of the measurements. The present study included a total of six fatty acid indicators, including PUFAs, MUFAs, SFAs, n-3 PUFAs, n-6 PUFAs, and n-3/n-6 PUFAs.

### Assessment of hypertension

The outcome measured in this study was the occurrence of hypertension. As previously described (31, 32), the UK Biobank collected data from health episode statistics and death certificates according to the International Classification of Diseases 10th and 9th Revision (ICD-10). Specifically, the relevant ICD-10 codes include I10, I11, I12, I13, I15, and O10, whereas the ICD-9 codes include 401 through 405. In the population excluding baseline hypertension, we also omitted participants who were diagnosed with hypertension and those on antihypertensive medications.

### Construction of the PRS for hypertension

Approximately 480,000 participants were genotyped in the UK Biobank (23, 33), and the details of the quality control and imputation procedures were performed as previously described (23, 33, 34). The PRS for hypertension was derived from single-nucleotide polymorphisms (SNPs) reported in a previous study (21, 32, 35). PRS calculations for hypertension were performed in this study via standard PRS (Category 301) data obtained from the UK Biobank, specifically field ID 26244 for hypertension, as per previous studies (32, 35). The participants were categorized as having low, intermediate, or high genetic risk of hypertension in noncases by tertiles, as described previously (31, 36, 37).

### Assessment of covariates

In accordance with previous studies (6, 23, 38–41), we determined the covariates that were considered in this analysis, including age, sex (male/female), race (white/Asian/black/other/missing), towns deprivation index (TDI), healthy diet (unhealthy/healthy/missing), body mass index (BMI) (<25 kg/m^2^/25 to 29.9 kg/m^2^/≥30 kg/m^2^, missing), alcohol consumption (daily or almost daily/three or four times a week/once or twice a week/one to three times a month/special occasion only/never/missing), smoking status (never/before/current/missing), diabetes status (yes/no/missing) and family history of disease (CVD/other diseases/missing). The authors adhered to the 2017 UK Physical Activity Guidelines to assess physical activity, which was determined by meeting the criteria of engaging in 150 min of walking or moderate activity or 75 min of vigorous activity per week (42). In accordance with a previous study (32), using recent dietary recommendations for cardiovascular health, we evaluated dietary quality, and the healthy level was defined as the intake of more than 5 dietary components (Supplementary Table S1). Weight data were collected from participants using a Tanita BC418MA body composition analyzer, and height measurements were obtained with a Seca 240 cm height measure. BMI was calculated using the formula: weight (kg)/height (m^2^). The diagnosis of diabetes was determined using the touchscreen questionnaire, which asked participants, “Has a doctor ever told you that you have diabetes?” The first 10 genetic principal components were further adjusted while the genetic data were analysed. Missing data for continuous variables and categorical variables were substituted by the mean values and missing indicator categories, respectively. Information about the covariates, including demographic characteristics and socioeconomic conditions, was obtained from the touchscreen questionnaire as described previously (25).

### Statistical analysis

Continuous variables and categorical variables are presented as the mean combined standard deviation and the number of cases combined percentage, respectively. For covariates, missing values for continuous variables were replaced with mean values, whereas missing data for categorical variables were addressed via dummy variables. A Cox proportional hazards model was used to estimate the hazard ratio (HR) and 95% confidence interval (CI) for hypertension according to quartiles or 1-SD increments of specific plasma FAs, which were included in the present study as percentages of total plasma FAs, which were more meaningful than absolute concentrations with regard to metabolic relationships, as previously described (43). Schoenfeld residuals were used to test the proportional hazard assumption. In the multivariate analysis of hypertension, we constructed Model 1 and Model 2, in which Model 1 was adjusted for age and sex, and Model 2 was adjusted for other confounding factors according to previous studies (6, 38–41), including race, TDI, physical activity, BMI, alcohol consumption, smoking status, diet score, diabetes status and family history of disease. We conducted restricted cubic spline (RCS) regression to evaluate the dose-response relationship between plasma FA levels and hypertension risk. A total of 195,250 participants were included in this study. The interaction between plasma fatty acids (FAs) and genetic risk factors was evaluated via two statistical measures: the relative excess risk due to interaction (RERI) and the attributable proportion due to interaction (AP). To identify subgroups susceptible to plasma FAs, we conducted stratified analyses by age (<60/≥60 years), sex (male vs. female), BMI (<25 kg/m^2^/25 to 29.9 kg/m^2^/≥30 kg/m^2^), physical activity (yes/no/unknown), smoking status (current/before/never), alcohol consumption (daily or almost daily/three or four times a week/once or twice a week/one to three times a month/special occasions only/never/missing) and economic level (Townsend deprivation index (TDI) <−3.22/−3.22 to −0.78/≥−0.78). Effect modification by these factors was tested via the heterogeneity test. In the sensitivity analyses, we additionally adjusted the usage of statins and excluded participants with a follow-up time of less than 2 years, those with CVD at baseline and nonwhite participants. Statistical analyses were performed via R software (version 4.2.1), and the *p*-values of all tests were two-sided, with values less than 0.05 considered statistically significant.

## Results

### Analysis of patient characteristics

A total of 195,250 participants were ultimately included in the present study. Table 1 and Supplementary Table S2 summarize the baseline characteristics of the participants and present the results stratified on the basis of the quantiles of PUFAs, MUFAs and SFAs. As shown in Table 1 and Supplementary Table S2, individuals with lower concentrations of PUFAs tended to be male, be current smokers, have higher BMIs, and have lower diet scores, whereas individuals with lower concentrations of MUFAs and SFAs tended to be female, never smokers, have lower BMIs, and have higher diet scores.

**Table 1:** Clinical characteristics by quantile of PUFAs (*n* = 195,819).

	PUFAs			
	Quantile 1	Quantile 2	Quantile 3	Quantile 4
	(*N* = 48,825)	(*N* = 48,817)	(*N* = 48,795)	(*N* = 48,813)
Age (years), mean (SD)	56.0 (8.05)	56.0 (8.10)	55.3 (8.14)	54.4 (8.23)
Sex, %				
Female	19,663 (40.3%)	27,212 (55.7%)	31,121 (63.8%)	31,826 (65.2%)
Male	29,162 (59.7%)	21,605 (44.3%)	17,674 (36.2%)	16,987 (34.8%)
Race, %				
White	47,355 (97.0%)	47,127 (96.5%)	46,627 (95.6%)	44,021 (90.2%)
Asian or Asian British	555 (1.1%)	631 (1.3%)	744 (1.5%)	1,443 (3.0%)
Black or Black British	128 (0.3%)	233 (0.5%)	410 (0.8%)	1,596 (3.3%)
Chinese	76 (0.2%)	91 (0.2%)	132 (0.3%)	346 (0.7%)
Mixed	232 (0.5%)	237 (0.5%)	305 (0.6%)	382 (0.8%)
Other ethnic group	262 (0.5%)	304 (0.6%)	369 (0.8%)	741 (1.5%)
Missing value	217 (0.4%)	194 (0.4%)	208 (0.4%)	284 (0.6%)
BMI, %				
Normal (<25 kg/m^2^)	8,983 (18.4%)	15,759 (32.3%)	21,814 (44.7%)	27,900 (57.2%)
Overweight (25 to 29.9 kg/m^2^)	23,375 (47.9%)	22,548 (46.2%)	20,436 (41.9%)	16,920 (34.7%)
Obesity (≥30 kg/m^2^)	16,295 (33.4%)	10,352 (21.2%)	6,414 (13.1%)	3,844 (7.9%)
Missing value	172 (0.4%)	158 (0.3%)	131 (0.3%)	149 (0.3%)
Smoke status, %				
Never	22,743 (46.6%)	26,400 (54.1%)	28,899 (59.2%)	31,565 (64.7%)
Previous	17,503 (35.8%)	16,184 (33.2%)	15,444 (31.7%)	14,325 (29.3%)
Current	8,305 (17.0%)	6,007 (12.3%)	4,247 (8.7%)	2,706 (5.5%)
Missing value	274 (0.6%)	226 (0.5%)	205 (0.4%)	217 (0.4%)
Alcohol consumption, %				
Daily or almost daily	10,842 (22.2%)	10,229 (21.0%)	9,537 (19.5%)	7,840 (16.1%)
Three or four times a week	11,161 (22.9%)	11,746 (24.1%)	12,121 (24.8%)	11,663 (23.9%)
Once or twice a week	12,444 (25.5%)	13,025 (26.7%)	13,328 (27.3%)	13,310 (27.3%)
One to three times a month	5,444 (11.2%)	5,290 (10.8%)	5,569 (11.4%)	5,885 (12.1%)
Special occasions only	5,326 (10.9%)	5,120 (10.5%)	4,958 (10.2%)	5,587 (11.4%)
Never	3,488 (7.1%)	3,315 (6.8%)	3,212 (6.6%)	4,400 (9.0%)
Missing value	120 (0.2%)	92 (0.2%)	70 (0.1%)	128 (0.3%)
Townsend deprivation index	−1.22 (3.11)	−1.49 (2.98)	−1.61 (2.92)	−1.50 (3.04)
Family history, %				
CVD	35,327 (72.4%)	35,492 (72.7%)	35,555 (72.9%)	35,327 (72.4%)
Other	12,849 (26.3%)	12,700 (26.0%)	12,647 (25.9%)	12,922 (26.5%)
No disease	649 (1.3%)	625 (1.3%)	593 (1.2%)	564 (1.2%)
Diet score				
Unhealthy	35,836 (73.4%)	33,979 (69.6%)	32,402 (66.4%)	29,645 (60.7%)
Healthy	10,236 (21.0%)	12,191 (25.0%)	13,814 (28.3%)	16,126 (33.0%)
Missing	2,753 (5.6%)	2,647 (5.4%)	2,579 (5.3%)	3,042 (6.2%)
Physical activity, %				
No	7,949 (16.3%)	6,861 (14.1%)	6,095 (12.5%)	5,862 (12.0%)
Yes	29,489 (60.4%)	30,950 (63.4%)	32,239 (66.1%)	33,379 (68.4%)
Unknown	11,387 (23.3%)	11,006 (22.5%)	10,461 (21.4%)	9,572 (19.6%)

### Associations between plasma FA levels and hypertension

The average follow-up time for this study was 12.48 years. Table 2 shows the associations between plasma fatty acid concentrations and hypertension. In Model 1, higher concentrations of SFAs and MUFAs were related to higher hypertension risk, and higher concentrations of PUFAs, n-6 PUFAs, n-3 PUFAs and the ratio of n-3 PUFAs to n-6 PUFAs were associated with lower hypertension risk. When all covariates were adjusted, the difference was still statistically significant (PUFAs: HR, 0.878; 95% CI, 0.868–0.888; MUFAs: HR, 1.137; 95% CI, 1.123–1.150; SFAs: HR, 1.086; 95% CI, 1.074–1.098; n-3 PUFAs: HR, 0.984; 95% CI, 0.973–0.995), which included race, physical activity, smoking status, alcohol consumption, BMI, TDI and diabetes, family history of CVD and diet score except n-3 PUFAs. RCS regression revealed similar results, except for the ratio of n-6 PUFAs to n-3 PUFAs, which was not statistically significant (Figure 1). Stratified analyses revealed that the effects of plasma FAs (per 1-SD) on hypertension were generally similar across different subgroups in terms of physical activity, TDI, smoking status and alcohol consumption. We detected heterogeneity among the subgroups according to sex, age and BMI, but the trends within the groups were consistent (Supplementary Table S3), and the female subgroup, nonelderly subgroup and normal-BMI subgroup seemed to be more sensitive to the effect of plasma FA levels on hypertension risk.

**Table 2:** Adjusted hazard ratio (HR) and 95% confidence interval (95% CI) of hypertension according to plasma FA exposure.

	Quartiles of plasma FA (% of total fatty acids)				*p* trend	HR (95% CI)^a^
	Quantile 1	Quantile 2	Quantile 3	Quantile 4		
PUFAs						
Model 1	1.00	0.753 (0.733, 0.774)	0.618 (0.600, 0.637)	0.541 (0.525, 0.559)	<0.001	0.789 (0.781, 0.797)
Model 2	1.00	0.864 (0.840, 0.888)	0.780 (0.757, 0.805)	0.720 (0.696, 0.745)	<0.001	0.878 (0.868, 0.888)
MUFAs						
Model 1	1.00	1.177 (1.137, 1.218)	1.452 (1.404, 1.500)	1.913 (1.852, 1.975)	<0.001	1.293 (1.279, 1.306)
Model 2	1.00	1.071 (1.034, 1.109)	1.195 (1.155, 1.237)	1.367 (1.320, 1.415)	<0.001	1.137 (1.123, 1.150)
SFAs						
Model 1	1.00	0.996 (0.965, 1.029)	1.100 (1.066, 1.135)	1.341 (1.301, 1.381)	<0.001	1.135 (1.123, 1.147)
Model 2	1.00	1.007 (0.975, 1.040)	1.086 (1.052, 1.121)	1.208 (1.171, 1.246)	<0.001	1.086 (1.074, 1.098)
n-6 PUFAs						
Model 1	1.00	1.009 (0.980, 1.039)	1.022 (0.992, 1.053)	1.001 (0.971, 1.033)	0.202	1.016 (1.004, 1.027)
Model 2	1.00	0.982 (0.954, 1.011)	0.978 (0.949, 1.008)	0.942 (0.912, 0.972)	0.018	0.991 (0.980, 1.003)
n-3 PUFAs						
Model 1	1.00	0.954 (0.925, 0.983)	0.915 (0.888, 0.943)	0.834 (0.808, 0.860)	0.002	0.931 (0.921, 0.941)
Model 2	1.00	0.988 (0.958, 1.019)	0.988 (0.958, 1.019)	0.968 (0.938, 0.999)	0.439	0.984 (0.973, 0.995)
n-3/n-6 ratio						
Model 1	1.00	0.991 (0.961, 1.022)	0.962 (0.933, 0.993)	0.917 (0.889, 0.946)	0.581	0.966 (0.956, 0.977)
Model 2	1.00	1.014 (0.983, 1.046)	1.016 (0.984, 1.048)	1.019 (0.988, 1.052)	0.394	0.999 (0.989, 1.010)

Model 1 was adjusted for age and sex (male/female). Model 2 included Model 1 plus race (white/Asian/black/other/missing), the TDI, a healthy diet (unhealthy/healthy/missing), physical activity (no/yes/unknown), body mass index (BMI) (<25 kg/m^2^, 25 to 29.9 kg/m^2^, ≥30 kg/m^2^, missing), alcohol consumption (daily or almost daily/three or four times a week/once or twice a week/one to three times a month/special occasion only/never/missing), smoking status (never/before/current/missing), diabetes status (yes/no/missing) and family history of disease (CVD/other diseases/missing).

a Per-SD of exposure.

### Associations of genetic risk with plasma FA levels and hypertension

We evaluated whether genetic risk factors affect the association between plasma FA and hypertension. A significant increase in hypertension risk across the deciles of the PRS was observed (*p* trend <0.001; Supplementary Table S4), and when the low PRS was used as the reference, hypertension risk was greater for those with intermediate genetic risk and high genetic risk (Model 1, intermediate HR, 1.334; 95% CI, 1.297–1.372; high HR, 1.751; 95% CI, 1.705–1.798; Model 2, intermediate HR, 1.356; 95% CI, 1.289–1.427; high HR, 1.329; 95% CI, 1.301–1.358) (Supplementary Table S5).

**Figure 2:**
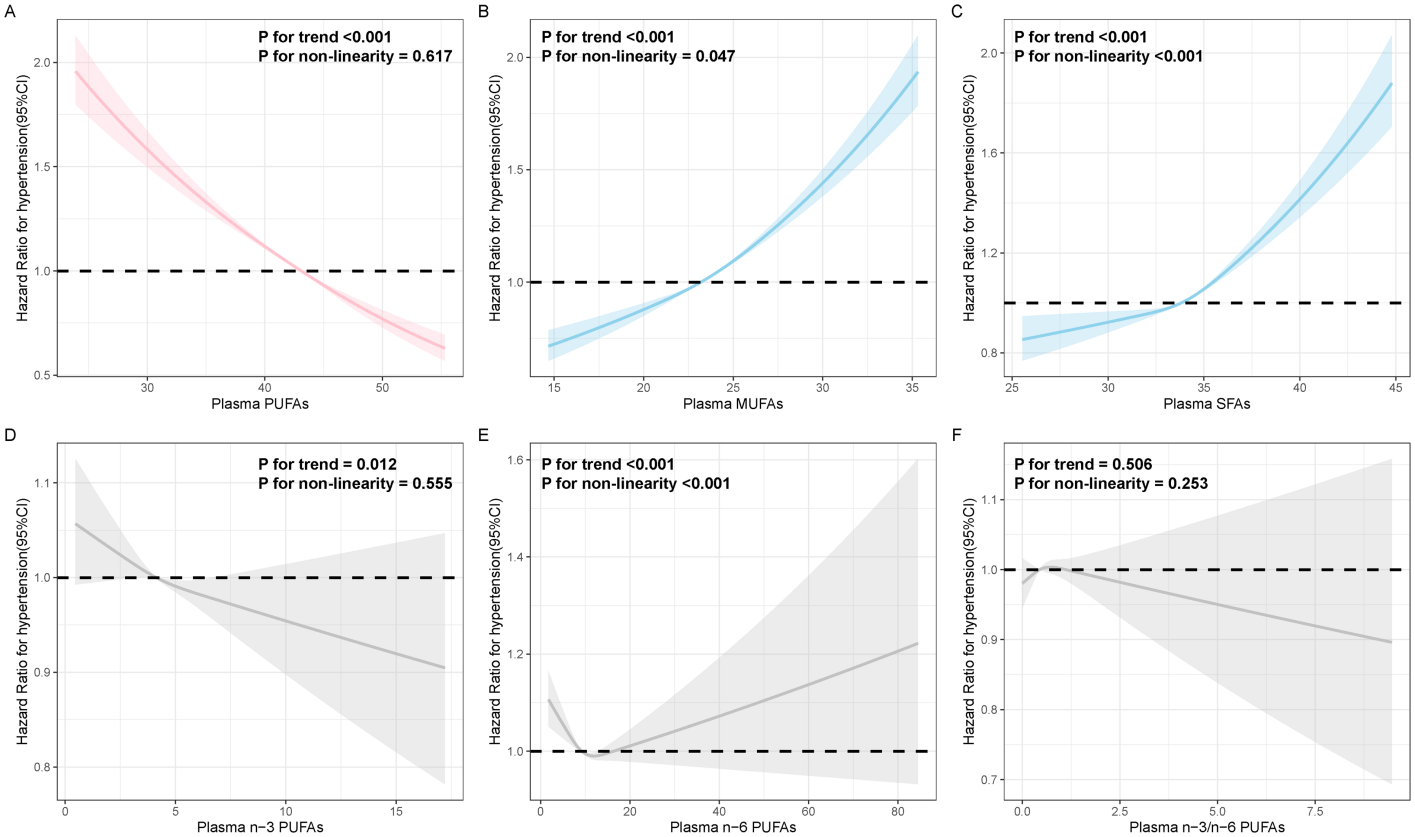
Dose-response relationships between plasma FA and hypertension risk. HRs for hypertension associated with plasma PUFAs **(A)**, MUFAs **(B)**, SFAs **(C)**, n-3 PUFAs **(D)**, n-6 PUFAs **(E)**, and the n-6/n-3 ratio **(F)** were estimated via restricted cubic-spline regression via Model 2.

**Figure 3:**
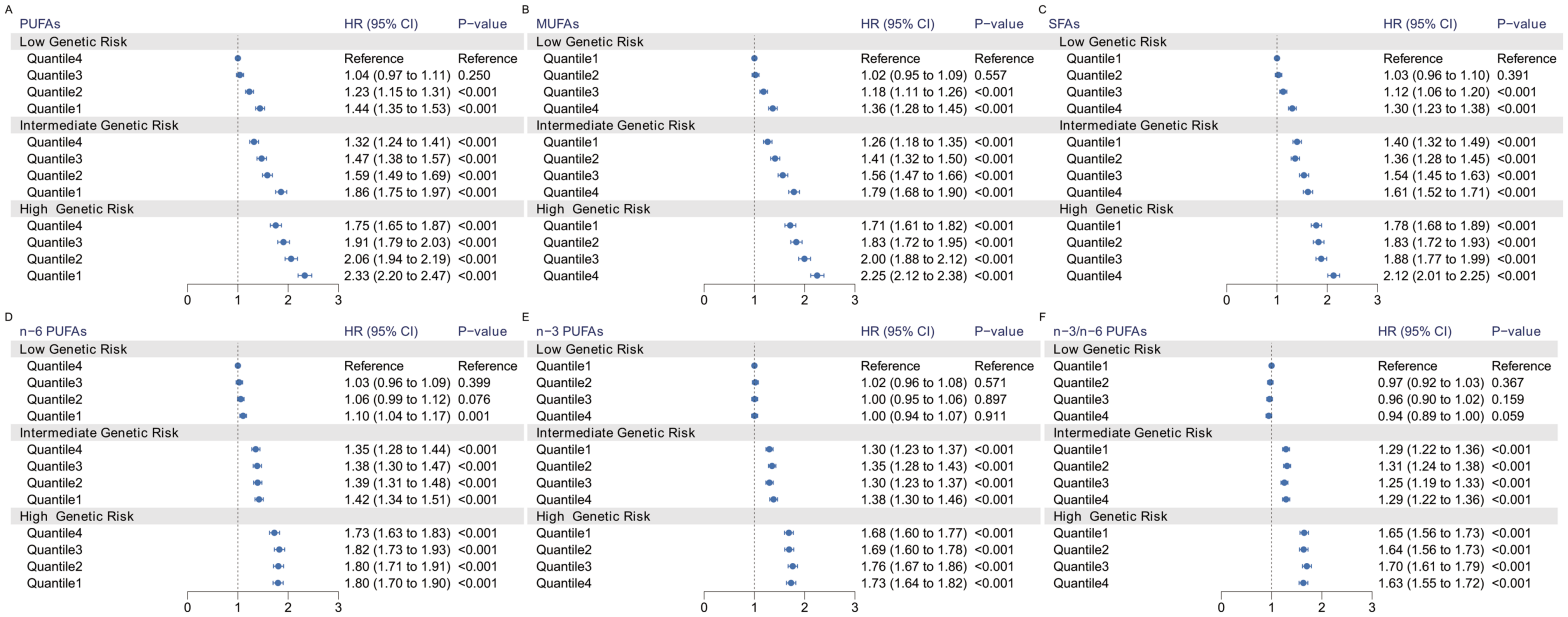
Risk of incident hypertension according to the concentration of plasma fatty acids and genetic risk categories. The HRs for hypertension according to PUFAs (A), MUFAs (B), SFAs (C), n-6 PUFAs (D), n-3 PUFAs (E), n-3/n-6 PUFAs (F) and polygenic risk score categories were estimated via Model 2 plus the genotyping batch and the first 4 genetic principal components.

We observed a significant joint association between plasma FA levels and genetic risk factors for hypertension, which exhibited a dose-response relationship. As shown in Figure 2, we investigated only the plasma FAs associated with a statistically significant risk of hypertension, including PUFAs, MUFAs, and SFAs. Compared with individuals with a low PRS and low plasma FA risk (quantile 4 as the reference for PUFAs and n-6 PUFAs, quantile 1 as the reference for MUFAs and SFAs), individuals with high genetic risk and high plasma FA risk had the highest risk of hypertension, as shown in Figure 3, in which the HR of the plasma MUFAs was 2.25 (95% CI: 2.12, 2.38), that of the PUFAs was 2.33 (95% CI: 2.20, 2.47) and that of the SFAs was 2.12 (95% CI: 2.01, 2.25). In addition, the RERIs and APs due to the additive interaction of genetic risk factors and plasma FAs were calculated (Table 3). Individuals with high genetic risk and high MUFA risk had the highest RERIs and APs [RERIs: 0.178 (95% CI: 0.062, 0.294), AP: 0.079 (95% CI: 0.027, 0.130)]. Therefore, we suggest that there would be 0.178 relative excess risk due to the additive interaction, accounting for 7.9% (95% CI: 2.7, 13.0%) of the hypertension risk. Moreover, significant interactions between other plasma FAs and genetic risk factors for hypertension were observed. Because of heterogeneity among subgroups according to sex, age and BMI for plasma FAs (per 1-SD) in hypertension patients, stratified analyses were also performed and revealed similar tendencies among the female subgroup, nonelderly subgroup and normal-BMI subgroup (Supplementary Figures S1–S6).

**Table 3:** RERIs and APs for additive interactions between shift work exposure and genetic categories.

		Intermediate risk		High risk		*p* for multiplicative interactions
		RERI (95% CI)	AP (95% CI)	RERI (95% CI)	AP (95% CI)	
PUFAs	Quantile 3	0.115 (0.008, 0.221)	0.077 (0.006, 0.149)	0.112 (−0.007, 0.230)	0.058 (−0.003, 0.120)	0.011
	Quantile 2	0.037 (−0.071, 0.144)	0.023 (−0.044, 0.091)	0.075 (−0.043, 0.194)	0.037 (−0.021, 0.094)	
	Quantile 1	0.098 (−0.010, 0.206)	0.052 (−0.005, 0.110)	0.146 (0.027, 0.265)	0.062 (0.012, 0.113)	
MUFAs	Quantile 1	0.118 (0.015, 0.221)	0.084 (0.010, 0.157)	0.092 (−0.024, 0.207)	0.050 (−0.013, 0.113)	0.034
	Quantile 2	0.116 (0.013, 0.219)	0.074 (0.008, 0.141)	0.096 (−0.019, 0.211)	0.048 (−0.010, 0.106)	
	Quantile 3	0.157 (0.053, 0.262)	0.087 (0.029, 0.145)	0.178 (0.062, 0.294)	0.079 (0.027, 0.130)	
SFAs	Quantile 1	−0.067 (−0.170, 0.036)	−0.049 (−0.124, 0.026)	0.018 (−0.093, 0.129)	0.010 (−0.050, 0.070)	0.038
	Quantile 2	0.013 (−0.090, 0.116)	0.009 (−0.058, 0.075)	−0.024 (−0.136, 0.087)	−0.013 (−0.072, 0.046)	
	Quantile 3	−0.093 (−0.197, 0.011)	−0.058 (−0.123, 0.007)	0.040 (−0.071, 0.151)	0.019 (−0.034, 0.071)	
n-6 PUFAs	Quantile 3	0.002 (−0.096, 0.100)	0.002 (−0.070, 0.073)	0.074 (−0.031, 0.179)	0.041 (−0.017, 0.099)	0.769
	Quantile 2	−0.024 (−0.122, 0.073)	−0.018 (−0.088, 0.053)	0.028 (−0.077, 0.133)	0.016 (−0.043, 0.074)	
	Quantile 1	−0.038 (−0.135, 0.059)	−0.027 (−0.095, 0.042)	−0.022 (−0.128, 0.083)	−0.012 (−0.071, 0.046)	
n-3 PUFAs	Quantile 3	0.037 (−0.054, 0.128)	0.027 (−0.039, 0.093)	−0.000 (−0.100, 0.099)	−0.000 (−0.058, 0.058)	0.364
	Quantile 2	0.003 (−0.088, 0.095)	0.002 (−0.067, 0.072)	0.081 (−0.018, 0.181)	0.046 (−0.010, 0.102)	
	Quantile 1	0.085 (−0.007, 0.177)	0.060 (−0.005, 0.125)	0.040 (−0.061, 0.141)	0.023 (−0.035, 0.081)	
n-3/n-6 PUFAs	Quantile 3	0.047 (−0.040, 0.134)	0.035 (−0.030, 0.101)	0.022 (−0.073, 0.117)	0.013 (−0.044, 0.071)	0.183
	Quantile 2	0.014 (−0.075, 0.102)	0.011 (−0.059, 0.080)	0.103 (0.008, 0.198)	0.060 (0.005, 0.116)	
	Quantile 1	0.061 (−0.028, 0.150)	0.047 (−0.021, 0.115)	0.037 (−0.060, 0.134)	0.023 (−0.037, 0.082)	

RERI, relative excess risk due to interaction; AP, attributable proportion due to interaction. PUFAs, n-6 PUFAs, n-3 PUFAs, and the n-3/n-6 PUFA ratio, with the fourth quartile serving as the reference group. For MUFAs and SFAs, the first quartile served as the reference group. Adjusted for age and sex (male/female), race (white/Asian/black/other/missing), Townsend deprivation index (TDI), healthy diet (unhealthy/healthy/missing), physical activity (no/yes/unknown), body mass index (BMI) (<25 kg/m^2^, 25 to 29.9 kg/m^2^, ≥30 kg/m^2^, missing), alcohol consumption (daily or almost daily/three or four times a week/once or twice a week/one to three times a month/special occasions only/never/missing), smoking status (never/before/current/missing), diabetes (yes/no/missing), family history of disease (CVD/other diseases/missing), and the first 10 genetic principal components.

### Sensitivity analyses

After further adjusting for the covariates of statin use at baseline and excluding participants with CVD at baseline, those with a follow-up time of less than 2 years and nonwhite participants, we conducted a sensitivity analysis. The analyses revealed that the observed associations between genetic risk, plasma FA concentration and hypertension risk were robust (Supplementary Figures S7–S14 and Supplementary Tables S6–S13). Restricted cubic spline regression and forest plots were used to visualize the relationships among genetic risk factors, plasma FA levels and hypertension (see Figure 2).

## Discussion

In the present study, on the basis of a prospective study of approximately 100,000 participants, we found that plasma FAs, including plasma PUFAs, MUFAs and SFAs, were associated with hypertension risk, in which concentrations of plasma PUFAs were related to a lower risk of hypertension, and plasma SFAs and MUFAs were related to a higher risk of hypertension. Importantly, interaction effects between plasma FA levels and genetic risk factors for hypertension were found, indicating that the relationship between plasma FA levels and the risk of hypertension could be modified by genetic risk factors. Among individuals with intermediate genetic risk, the associations between quantile 3 plasma MUFAs and high genetic risk of hypertension were the strongest positive. The present study, which used a large-scale sample prospective cohort study, provides remarkable insight into additive interactions between plasma FAs and hypertension PRS with respect to hypertension risk.

Previous work has confirmed the relationship between plasma FA exposure and hypertension. However, the conclusions have not been consistent. The Atherosclerosis Risk in Communities (ARIC) study (12) showed that the odds ratio (OR) estimates and 95% CI of incident hypertension for an interquartile increment of a fatty acid were MUFAs [1.11 (0.96, 1.28)], PUFAs [0.88 (0.75, 1.02)], SFAs [1.15 (0.97, 1.36)], 22:6n-3 [1.20 (1.04, 1.37)] and 20:5n-3 [1.16 (1.04, 1.28)], whereas the genetic and phenotypic determinants of blood pressure and other cardiovascular risk factors (GAPP) (13) showed that higher docosahexaenoic acid (DHA, 22:6n-3) and eicosapentaenoic acid (EPA, 20:5n-3) were correlated with lower blood pressure, and a similar result was observed among Black South African adults (14). In addition, conflicts have also been reported among other plasma FAs, such as SFAs and MUFAs. Yang et al. (15) reported that lower proportions of 14:0, 16:0 and 16:1n-7 were beneficial for increasing blood pressure, and Zheng et al. (12) reported that the risk of hypertension and SFAs was 2.01 (1.05, 2.98), whereas the ARIC reported a negative relationship with hypertension. The present study, which used a large prospective cohort study, strengthened the association with hypertension risk. Metabolic exposure to MUFAs had the strongest relationship with increased hypertension risk, followed by SFAs, and PUFAs and n-6 PUFAs had similar associations with decreased hypertension risk. However, as one of the limitations of this study, we cannot estimate the similarities and differences in the metabonomic components of different plasma FAs, such as EPA.

Some studies have previously explored the relationships between plasma FA levels and hypertension or between genetic risk factors and hypertension; however, to our knowledge, the present study is the first to explore additive interactions between plasma FA levels and genetic risk factors for hypertension. We revealed that the risk of hypertension associated with plasma FAs was increased by genetic risk factors, which indicated that approximately 15–55% of the risk of hypertension could be attributed to additive interactions, and the additive interaction between quantile 1 MUFAs and intermediate risk was the most significant. In light of these findings, the possibility that plasma FAs modify the influence of genetic susceptibility on hypertension risk could be speculated.

Moreover, our findings have highlighted the public health implications for the prevention of hypertension. Hypertension constitutes a major disease burden worldwide, and the recommendations of blood pressure guidelines mention that changing unhealthy lifestyles, such as weekly aerobic exercise, a DASH diet, ideal weight and moderate alcohol consumption, can be effective measures for preventing hypertension (5, 44). However, the success of maintaining a healthy lifestyle depends mostly on the compliance of participants, and many factors may affect persistence, including biological, behavioral, psychosocial and environmental factors (45–47). In addition, previous studies reported that genetic risk, such as variants of the AGT gene encoding angiotensinogen, which plays a role in the renin-angiotensin system (48–50), may vary across ethnicities, indicating the need for tailored prevention programs and precision medicine. To date, the combined preventive effects of plasma FAs in individuals at high genetic risk for hypertension have not been investigated. To fill this gap, our study may provide new evidence that combining plasma FA levels and the genetic risk of hypertension could identify individuals with high hypertension risk and reduce the control cost.

Previous experimental mechanism studies have corroborated our findings. Oleic acid, a MUFA, has been shown in earlier studies to generate mitochondrial reactive oxygen species (51) and decrease cellular nitric oxide synthase activity, which contributes to vascular endothelial cell dysfunction. Similarly, palmitic acid, a saturated fatty acid, has been demonstrated to activate the p38/JNK pathway through the promotion of reactive oxygen species production, leading to the aging and dysfunction of vascular endothelial cells (52). This dysfunction is significantly associated with the onset and progression of hypertension. Additionally, EPA, a PUFA, has been reported in prior studies to mitigate renal oxidative stress by stimulating Nrf-2 and regulating interleukin (IL)-6 to enhance the anti-inflammatory response, thereby influencing systolic blood pressure (53).

The strengths of the study are as follows. First, and most importantly, we included a large sample size that was obtained from multiple centers and used uniform data collection protocols, including detailed demographic and lifestyle information. In addition, the biochemistry assays and assessment of metabolomics were performed in accordance with the internationally recognized standards for testing.

### Limitations of the study

The present study is not without limitations. First, the UK Biobank used NMR to analyse the metabolomics characteristics of participants. Although NMR can qualitatively measure known and unknown compounds, with only a small portion of the sample having the characteristics of noninvasive, nondestructive, highly repeatable and quantitative capabilities (54–56), the number of serum/plasma metabolites analysed by NMR is much lower than the actual number of metabolites in the actual sample (57), and the relative sensitivity is low (56). The attenuation caused by the combination of metabolites with serum/plasma may cause the concentration of many metabolites detected to be seriously underestimated (58, 59), thus affecting the analysis of plasma FAs and hypertension. Additionally, most participants in the UK Biobank were Europeans, which limits the applicability to those who are European white. The low participation rate of only 5.5% for the UK Biobank may lead to selection bias (60). Finally, selection bias, referred to as “healthy volunteers,” may also limit the representativeness of the present study (60).

## Conclusion

In this cohort of adults from the United Kingdom, plasma FA levels were associated with hypertension risk in a dose-response manner. These findings also suggest that the relationship between plasma FA levels and the risk of hypertension could be modified by genetic risk factors, which provides new insight into the relationships of plasma FA levels and hypertension PRS with hypertension risk.

## Data Availability

Publicly available datasets were analyzed in this study. This data can be found here: all UK Biobank information is available online on the webpage www.ukbiobank.ac.uk/. Data access is available through applications.

## Supplementary material

SUPPLEMENTARY FIGURE S1 Risk of incident hypertension according to concentration of plasma fatty acids and genetic risk categories of female subgroup. The HRs for hypertension according to PUFAs **(A)**, MUFAs **(B)**, SFAs **(C)**, n-6 PUFAs **(D)**, n-3 PUFAs **(E)**, n-3/n-6 PUFAs **(F)**, and polygenic risk score categories were estimated by using model 2 plus the first 10 genetic principal components.

SUPPLEMENTARY FIGURE S2 Risk of incident hypertension according to concentration of plasma fatty acids and genetic risk categories of male subgroup. The HRs for hypertension according to PUFAs **(A)**, MUFAs **(B)**, SFAs **(C)**, n-6 PUFAs **(D)**, n-3 PUFAs **(E)**, n-3/n-6 PUFAs **(F)**, and polygenic risk score categories were estimated by using model 2 plus the first 10 genetic principal components.

SUPPLEMENTARY FIGURE S3 Risk of incident hypertension according to concentration of plasma fatty acids and genetic risk categories of <60 subgroup. The HRs for hypertension according to PUFAs **(A)**, MUFAs **(B)**, SFAs **(C)**, n-6 PUFAs **(D)**, n-3 PUFAs **(E)**, n-3/n-6 PUFAs **(F)**, and polygenic risk score categories were estimated by using model 2 plus the first 10 genetic principal components.

SUPPLEMENTARY FIGURE S4 Risk of incident hypertension according to concentration of plasma fatty acids and genetic risk categories of ≥60 subgroup. The HRs for hypertension according to PUFAs **(A)**, MUFAs **(B)**, SFAs **(C)**, n-6 PUFAs **(D)**, n-3 PUFAs **(E)**, n-3/n-6 PUFAs **(F)**, and polygenic risk score categories were estimated by using model 2 plus the first 10 genetic principal components.

SUPPLEMENTARY FIGURE S5 Risk of incident hypertension according to concentration of plasma fatty acids and genetic risk categories of normal-BMI subgroup. The HRs for hypertension according to PUFAs **(A)**, MUFAs **(B)**, SFAs **(C)**, n-6 PUFAs **(D)**, n-3 PUFAs **(E)**, n-3/n-6 PUFAs **(F)**, and polygenic risk score categories were estimated by using model 2 plus the first 10 genetic principal components.

SUPPLEMENTARY FIGURE S6 Risk of incident hypertension according to concentration of plasma fatty acids and genetic risk categories of overweight-obesity BMI subgroup. The HRs for hypertension according to PUFAs **(A)**, MUFAs **(B)**, SFAs **(C)**, n-6 PUFAs **(D)**, n-3 PUFAs **(E)**, n-3/n-6 PUFAs **(F)**, and polygenic risk score categories were estimated by using model 2 plus the first 10 genetic principal components.

SUPPLEMENTARY FIGURE S7 Dose-response relationships between plasma FA and hypertension risk. HRs for hypertension associated with plasma PUFAs **(A)**, MUFAs **(B)**, SFAs **(C)**, n-3 PUFAs **(D)**, n-6 PUFAs **(E)**, and n-3/n-6 ratio **(F)** were estimated by restricted cubic-spline regression using model 2 additionally adjusted stains.

SUPPLEMENTARY FIGURE S8 Dose-response relationships between plasma FA and hypertension risk. HRs for hypertension associated with plasma PUFAs **(A)**, MUFAs **(B)**, SFAs **(C)**, n-3 PUFAs **(D)**, n-6 PUFAs **(E)**, and n-3/n-6 ratio **(F)** were estimated by restricted cubic-spline regression using model 2 excluding participants with were any type of cardiovascular disease at baseline in the UK Biobank.

SUPPLEMENTARY FIGURE S9 Dose-response relationships between plasma FA and hypertension risk. HRs for hypertension associated with plasma PUFAs **(A)**, MUFAs **(B)**, SFAs **(C)**, n-3 PUFAs **(D)**, n-6 PUFAs **(E)**, and n-3/n-6 ratio **(F)** were estimated by restricted cubic-spline regression using model 2 excluding participants with follow-up time of less than 2 years in the UK Biobank.

SUPPLEMENTARY FIGURE S10 Dose-response relationships between plasma FA and hypertension risk. HRs for hypertension associated with plasma PUFAs **(A)**, MUFAs **(B)**, SFAs **(C)**, n-3 PUFAs **(D)**, n-6 PUFAs **(E)**, and n-3/n-6 ratio **(F)** were estimated by restricted cubic-spline regression using model 2 only race White participants in the UK Biobank.

SUPPLEMENTARY FIGURE S11 Risk of incident hypertension according to concentration of plasma fatty acids and genetic risk categories. The HRs for hypertension according to PUFAs **(A)**, MUFAs **(B)**, SFAs **(C)**, n-6 PUFAs **(D)**, and polygenic risk score categories were estimated by using model 2 additionally adjusted stains plus the first 10 genetic principal components.

SUPPLEMENTARY FIGURE S12 Risk of incident hypertension according to concentration of plasma fatty acids and genetic risk categories. The HRs for hypertension according to PUFAs **(A)**, MUFAs **(B)**, SFAs **(C)**, n-6 PUFAs **(D)**, n-3 PUFAs **(E)**, n-3/n-6 PUFAs **(F)**, and polygenic risk score categories were estimated by using model 2 plus the first 10 genetic principal components excluding participants with were any type of cardiovascular disease at baseline in the UK Biobank.

SUPPLEMENTARY FIGURE S13 Risk of incident hypertension according to concentration of plasma fatty acids and genetic risk categories. The HRs for hypertension according to PUFAs **(A)**, MUFAs **(B)**, SFAs **(C)**, n-6 PUFAs **(D)**, n-3 PUFAs **(E)**, n-3/n-6 PUFAs **(F)**, and polygenic risk score categories were estimated by using model 2 plus the first 10 genetic principal components excluding participants with follow-up time of less than 2 years in the UK Biobank.

SUPPLEMENTARY FIGURE S14 Risk of incident hypertension according to concentration of plasma fatty acids and genetic risk categories. The HRs for hypertension according to PUFAs **(A)**, MUFAs **(B)**, SFAs **(C)**, n-6 PUFAs **(D)**, n-3 PUFAs **(E)**, n-3/n-6 PUFAs **(F)**, and polygenic risk score categories were estimated by using model 2 plus the first 10 genetic principal components only race White participants in the UK Biobank.
