# Supplementary figures and images for: Circulating fatty acids, genetic susceptibility and hypertension: a prospective cohort study
Source: Front Nutr. 2024 Oct 31;11:1454364. doi: 10.3389/fnut.2024.1454364 (PMC11562856; doi:10.3389/fnut.2024.1454364)

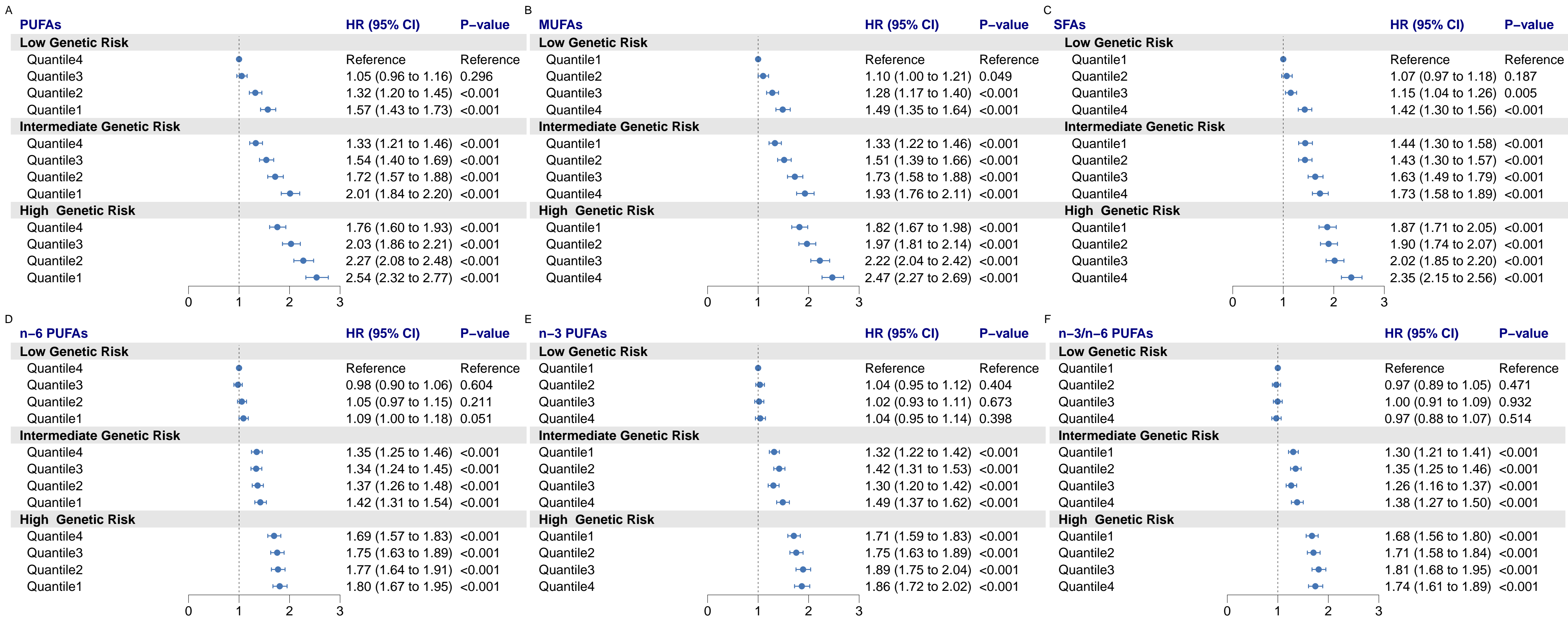

Supplement: SUPPLEMENTARY FIGURE S1 — Risk of incident hypertension according to concentration of plasma fatty acids and genetic risk categories of female subgroup. The HRs for hypertension according to PUFAs (A), MUFAs (B), SFAs (C), n-6 PUFAs (D), n-3 PUFAs (E), n-3/n-6 PUFAs (F), and polygenic risk score categories were estimated by using model 2 plus the first 10 genetic principal components. [file Image_1.PDF]

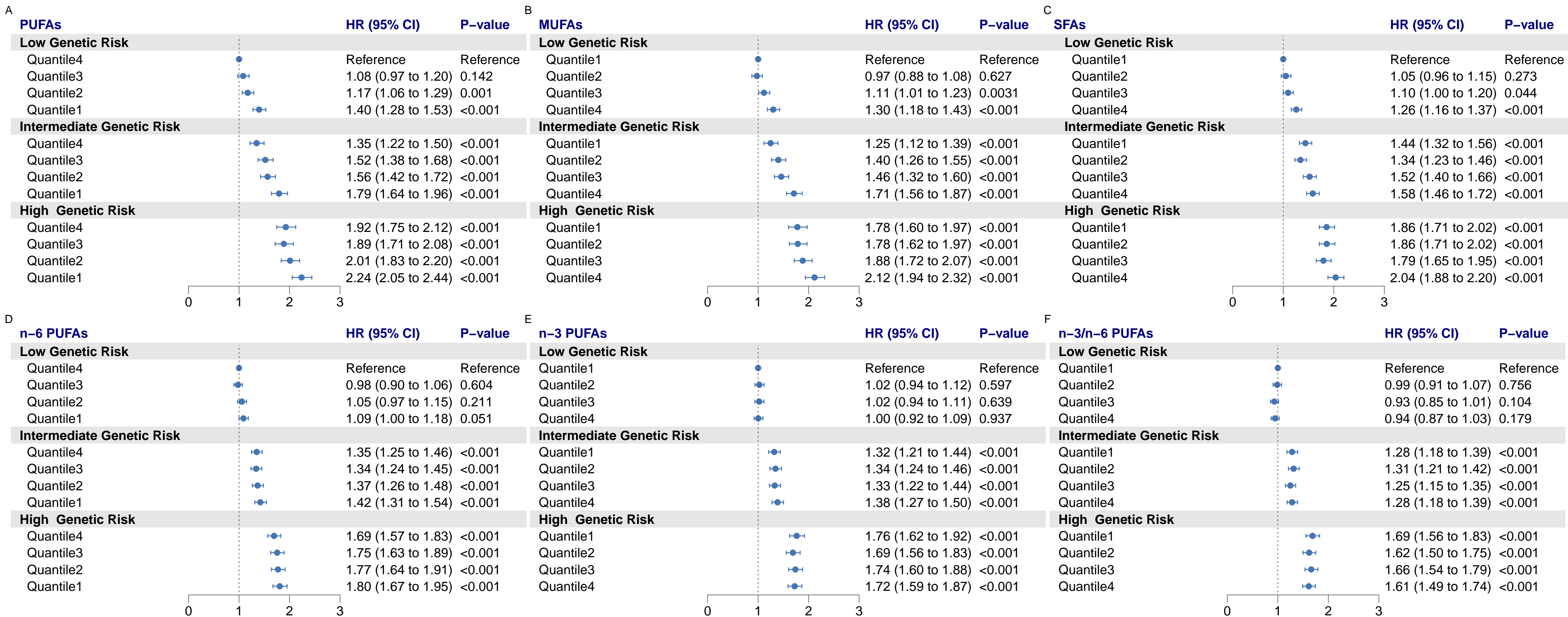

Supplement: SUPPLEMENTARY FIGURE S2 — Risk of incident hypertension according to concentration of plasma fatty acids and genetic risk categories of male subgroup. The HRs for hypertension according to PUFAs (A), MUFAs (B), SFAs (C), n-6 PUFAs (D), n-3 PUFAs (E), n-3/n-6 PUFAs (F), and polygenic risk score categories were estimated by using model 2 plus the first 10 genetic principal components. [file Image_2.PDF]

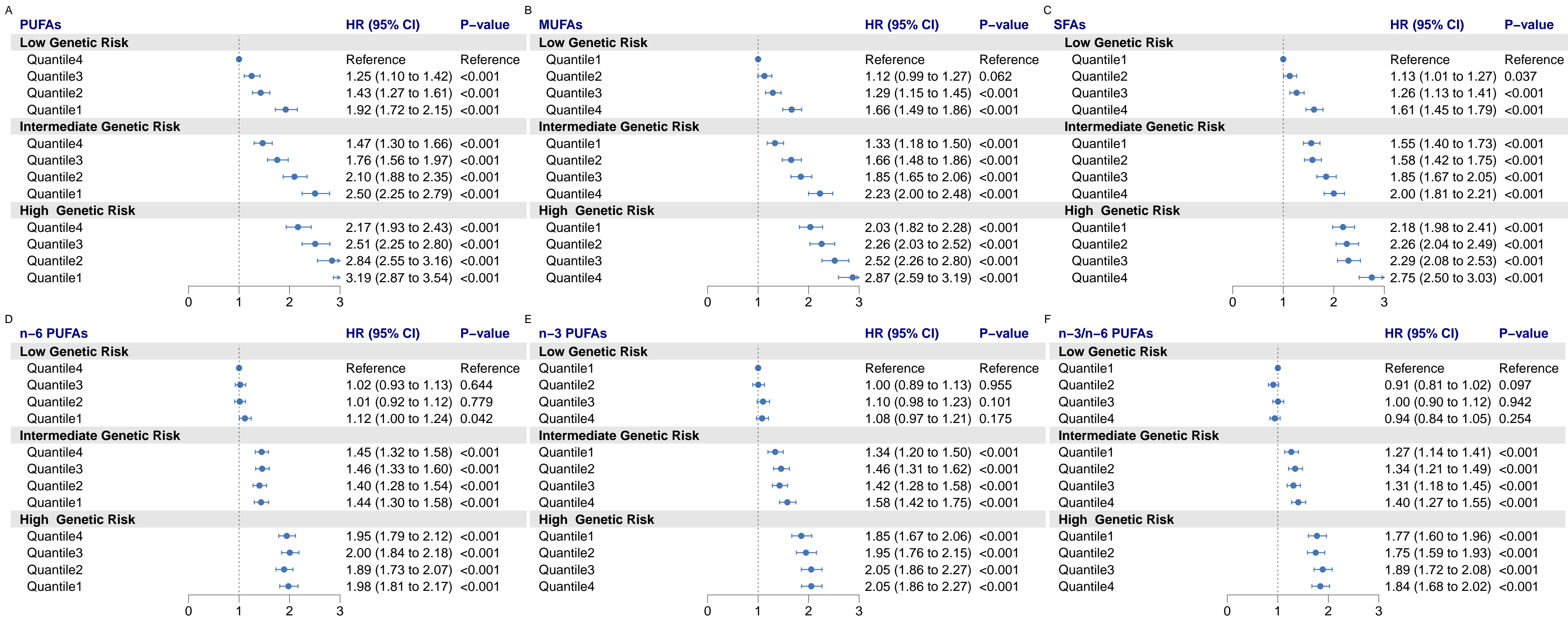

Supplement: SUPPLEMENTARY FIGURE S3 — Risk of incident hypertension according to concentration of plasma fatty acids and genetic risk categories of <60 subgroup. The HRs for hypertension according to PUFAs (A), MUFAs (B), SFAs (C), n-6 PUFAs (D), n-3 PUFAs (E), n-3/n-6 PUFAs (F), and polygenic risk score categories were estimated by using model 2 plus the first 10 genetic principal components. [file Image_3.PDF]

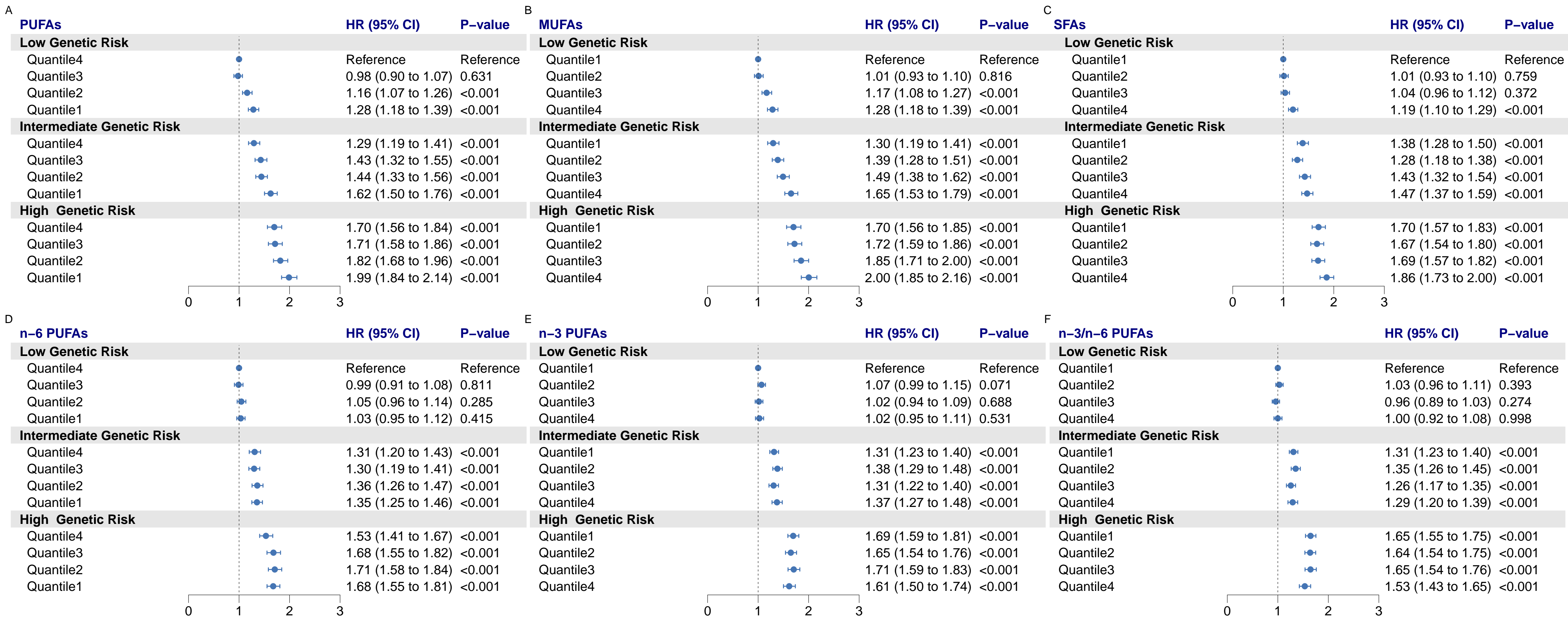

Supplement: SUPPLEMENTARY FIGURE S4 — Risk of incident hypertension according to concentration of plasma fatty acids and genetic risk categories of ≥60 subgroup. The HRs for hypertension according to PUFAs (A), MUFAs (B), SFAs (C), n-6 PUFAs (D), n-3 PUFAs (E), n-3/n-6 PUFAs (F), and polygenic risk score categories were estimated by using model 2 plus the first 10 genetic principal components. [file Image_4.PDF]

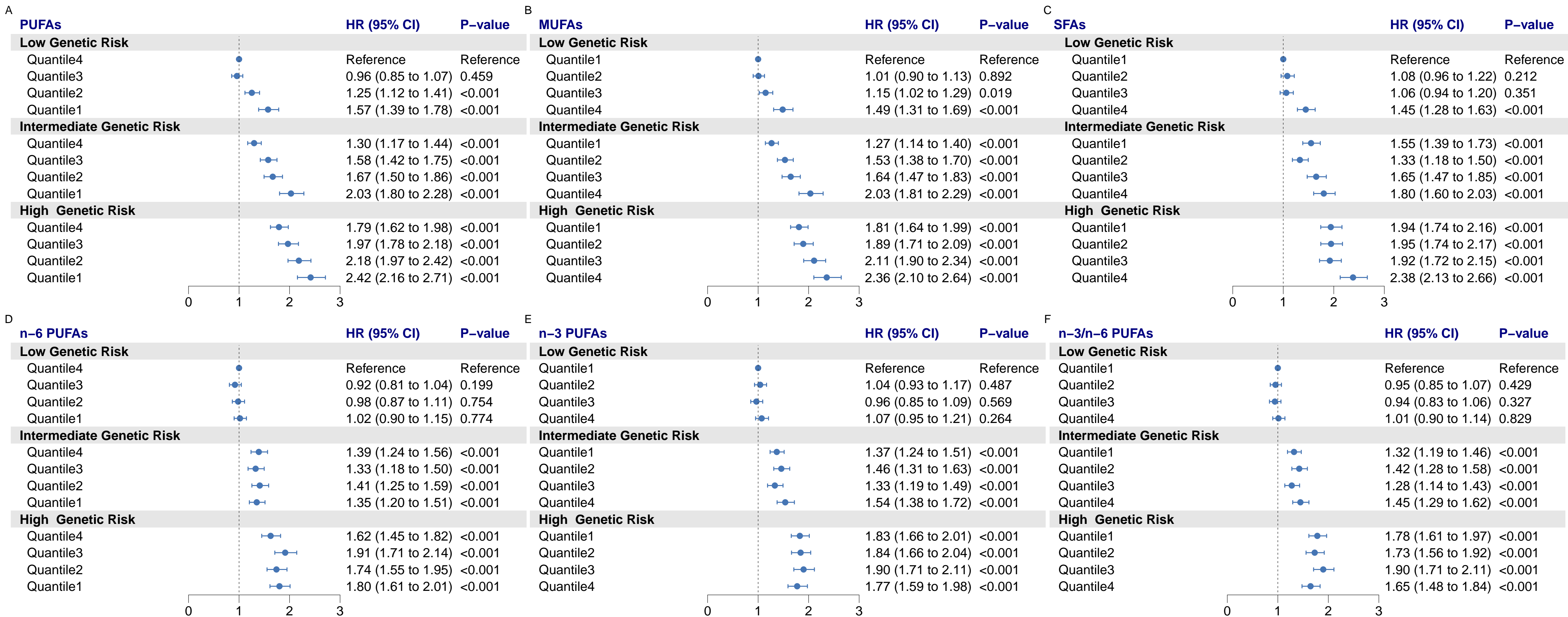

Supplement: SUPPLEMENTARY FIGURE S5 — Risk of incident hypertension according to concentration of plasma fatty acids and genetic risk categories of normal-BMI subgroup. The HRs for hypertension according to PUFAs (A), MUFAs (B), SFAs (C), n-6 PUFAs (D), n-3 PUFAs (E), n-3/n-6 PUFAs (F), and polygenic risk score categories were estimated by using model 2 plus the first 10 genetic principal components. [file Image_5.PDF]

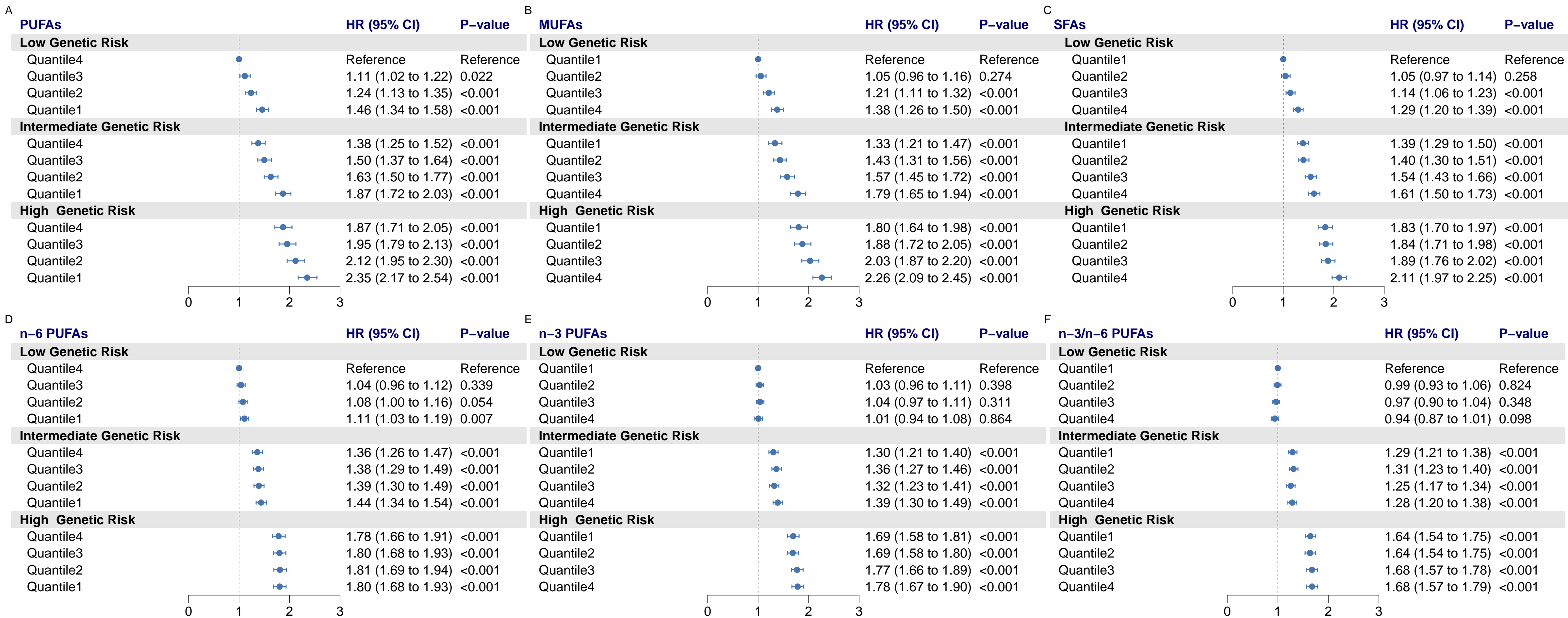

Supplement: SUPPLEMENTARY FIGURE S6 — Risk of incident hypertension according to concentration of plasma fatty acids and genetic risk categories of overweight-obesity BMI subgroup. The HRs for hypertension according to PUFAs (A), MUFAs (B), SFAs (C), n-6 PUFAs (D), n-3 PUFAs (E), n-3/n-6 PUFAs (F), and polygenic risk score categories were estimated by using model 2 plus the first 10 genetic principal components. [file Image_6.PDF]

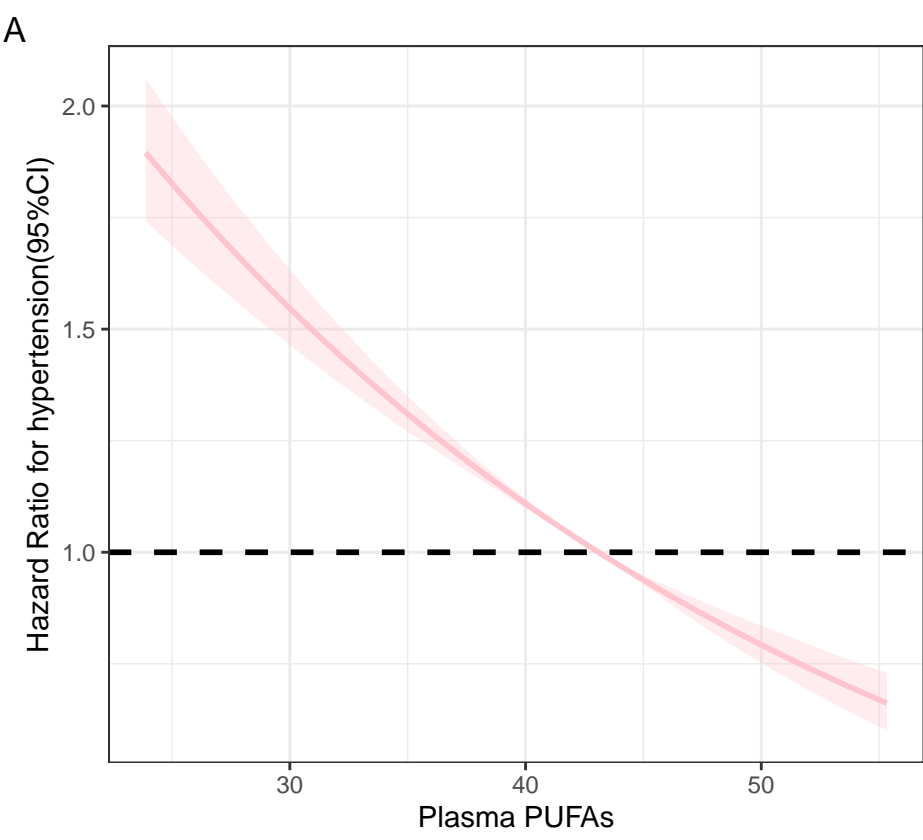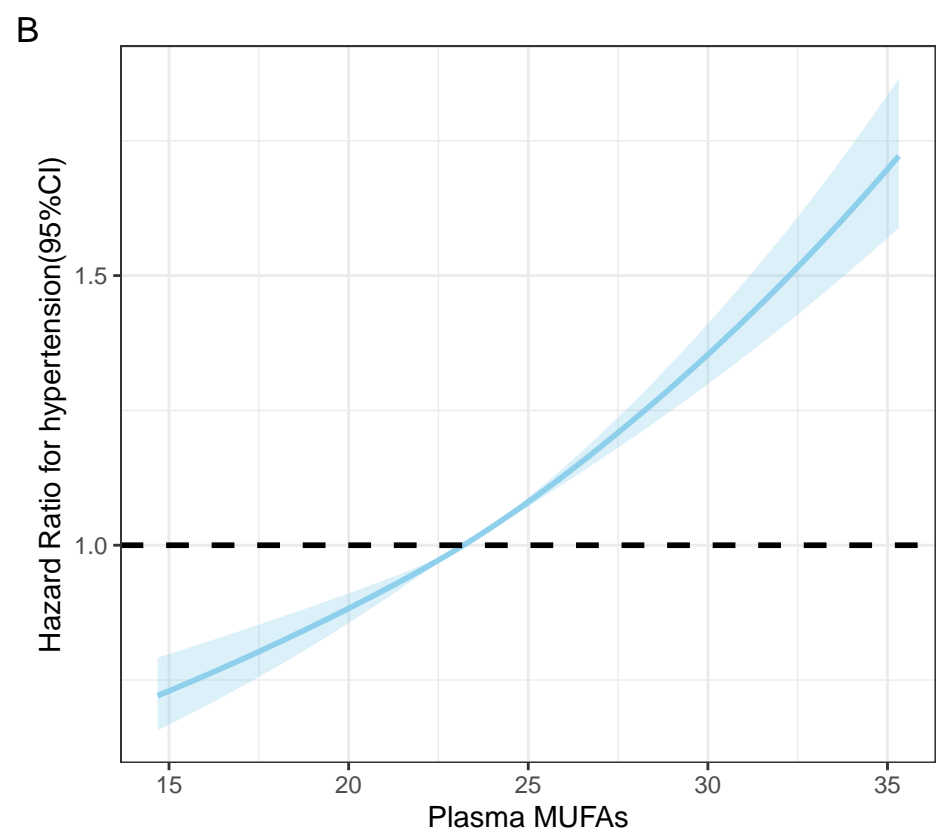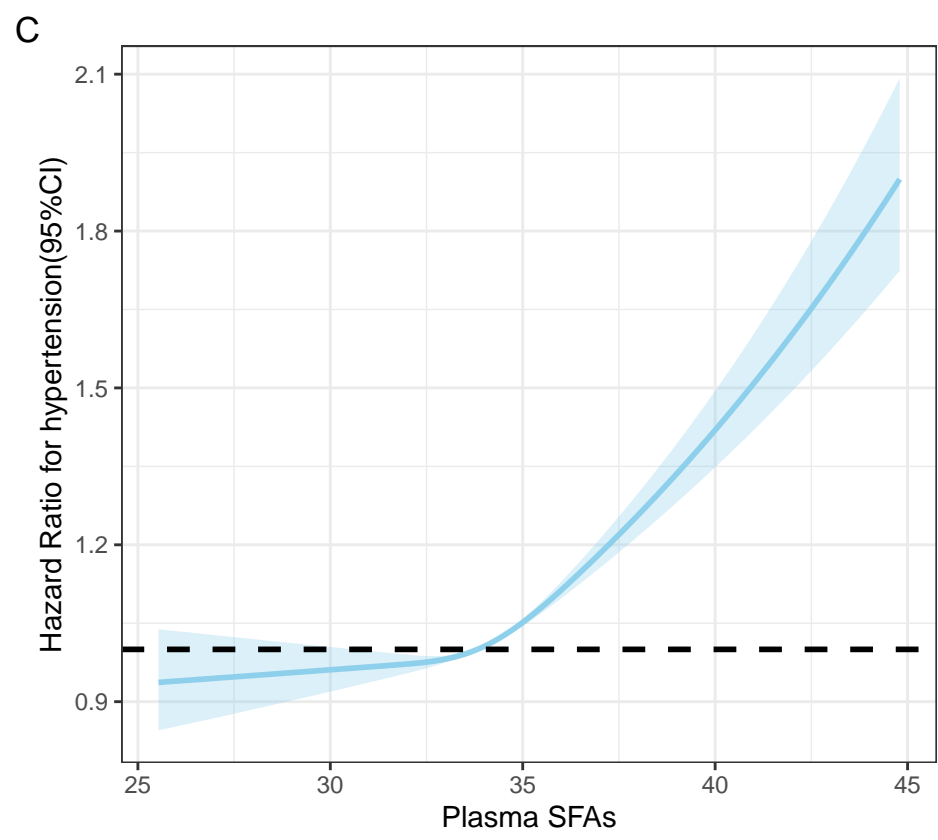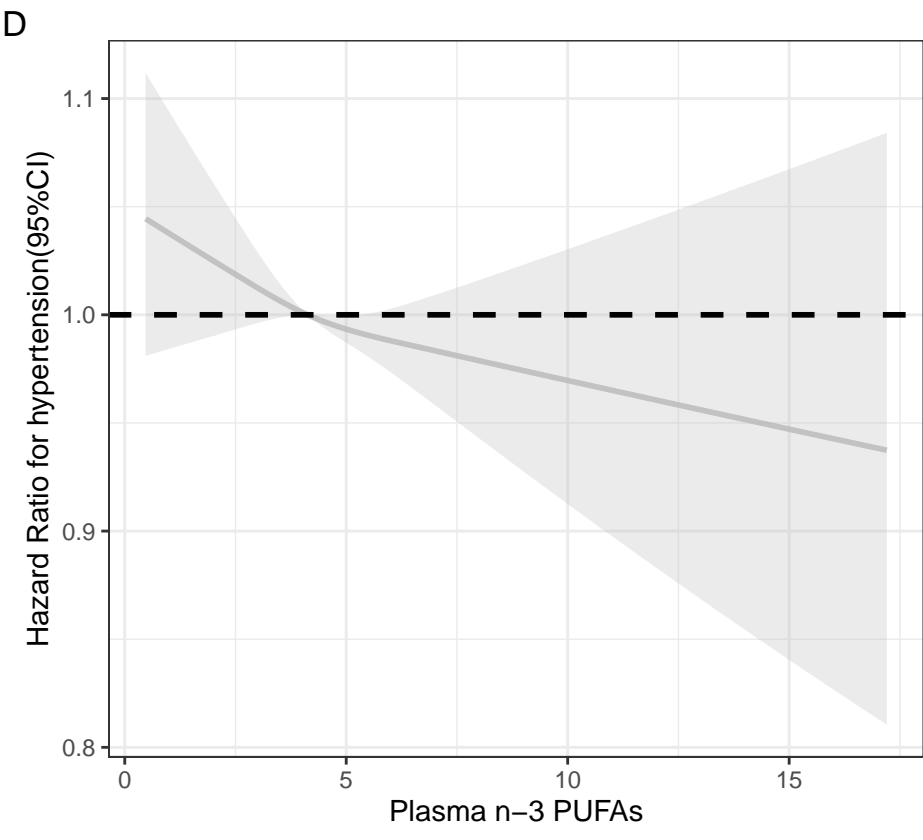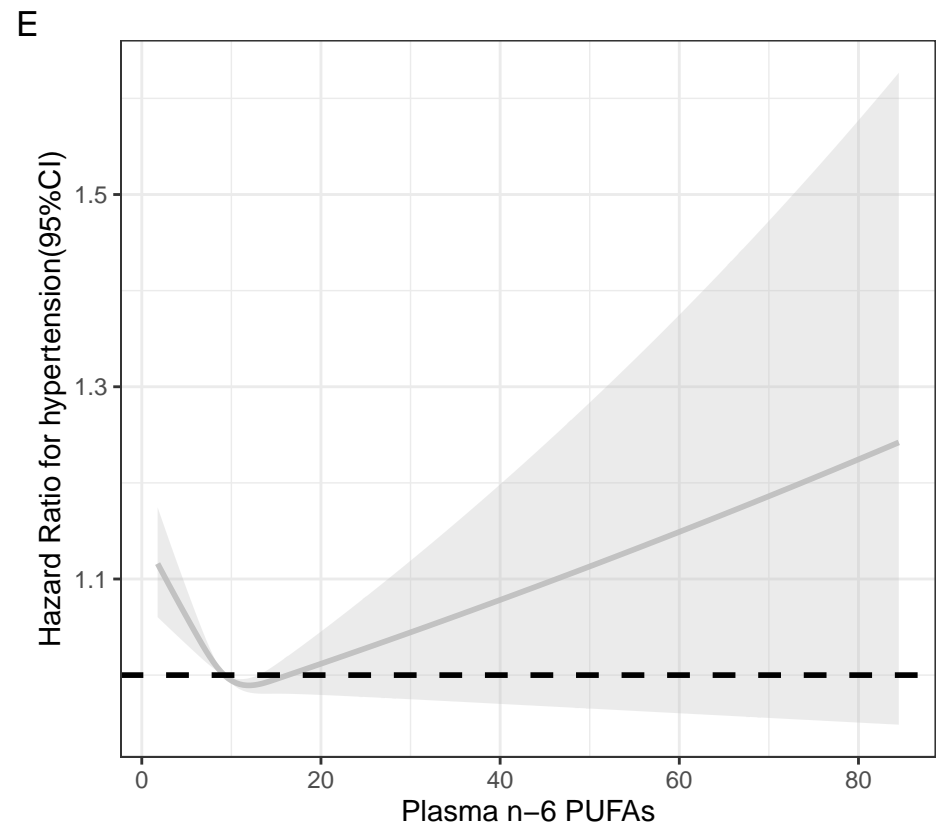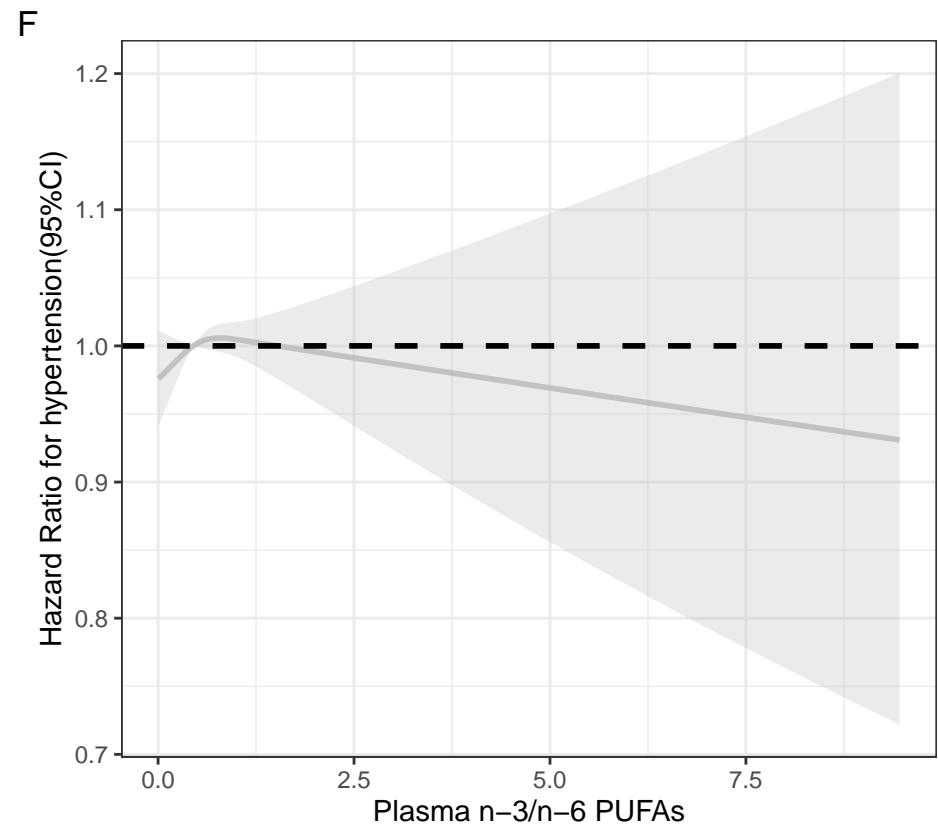

Supplement: SUPPLEMENTARY FIGURE S7 — Dose-response relationships between plasma FA and hypertension risk. HRs for hypertension associated with plasma PUFAs (A), MUFAs (B), SFAs (C), n-3 PUFAs (D), n-6 PUFAs (E), and n-3/n-6 ratio (F) were estimated by restricted cubic-spline regression using model 2 additionally adjusted stains. [file Image_7.PDF]

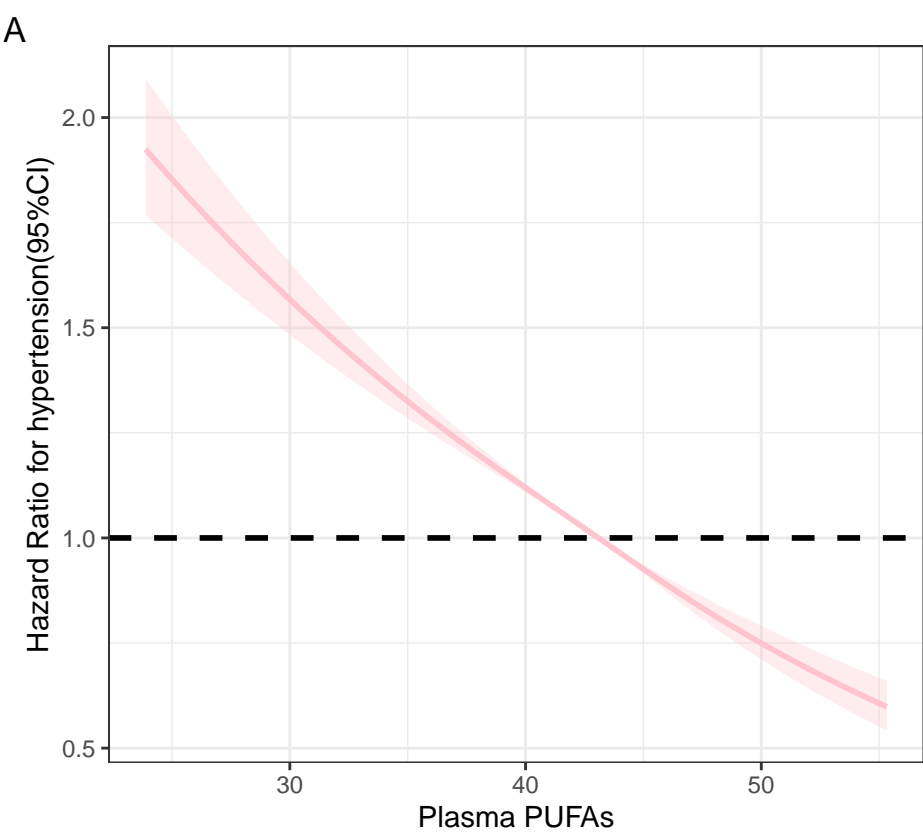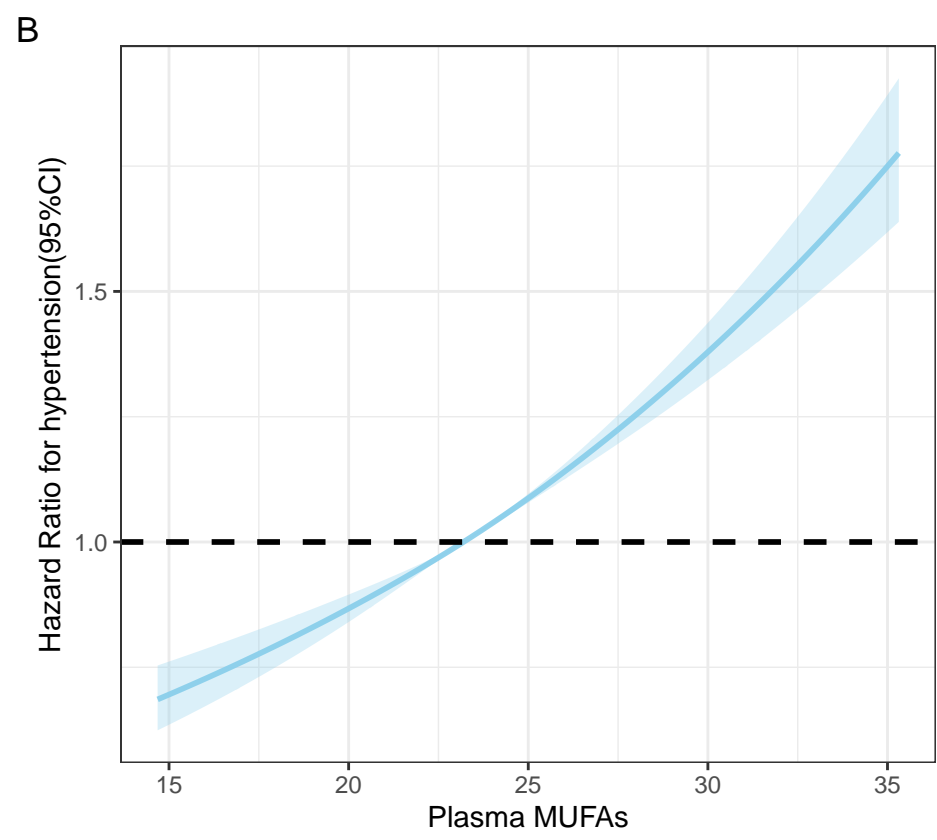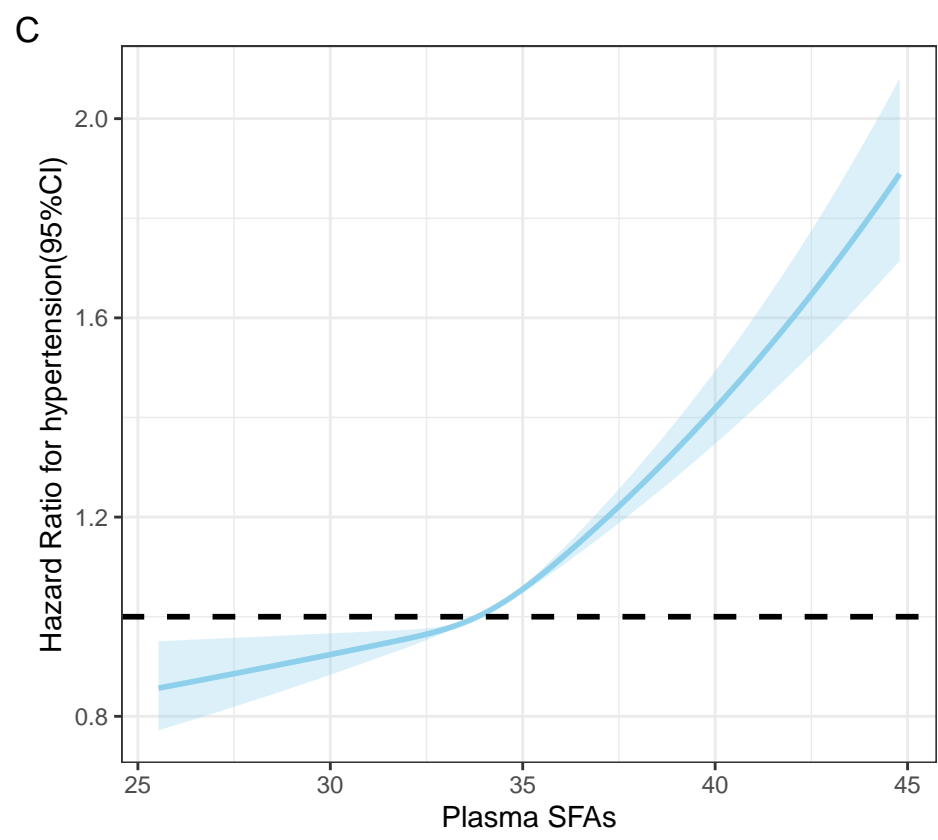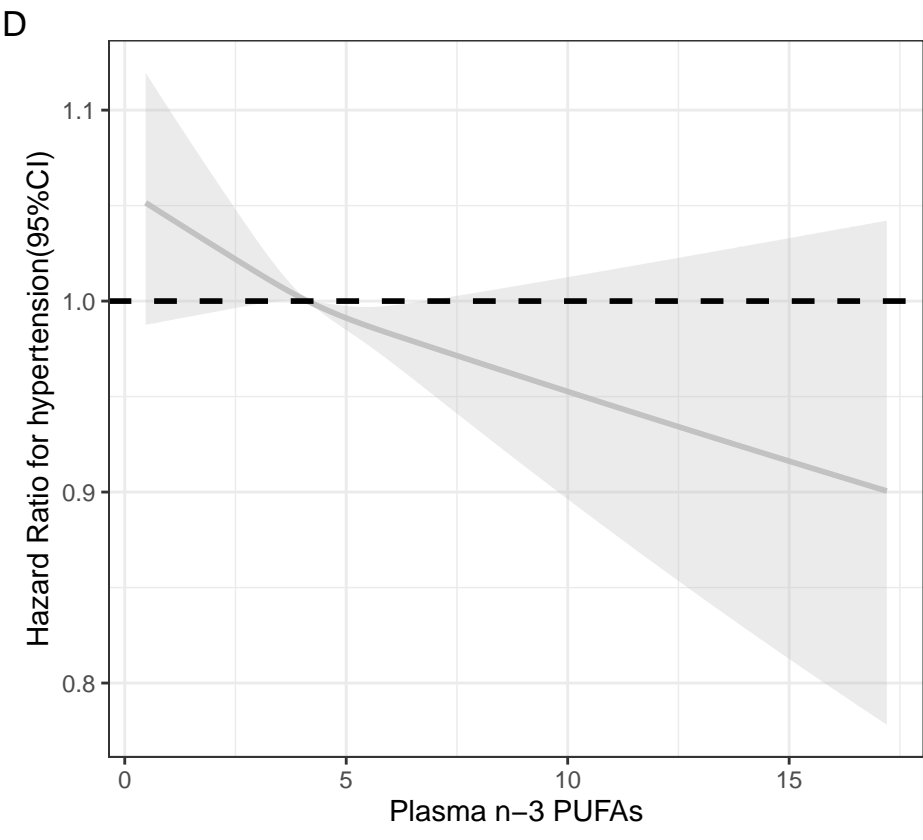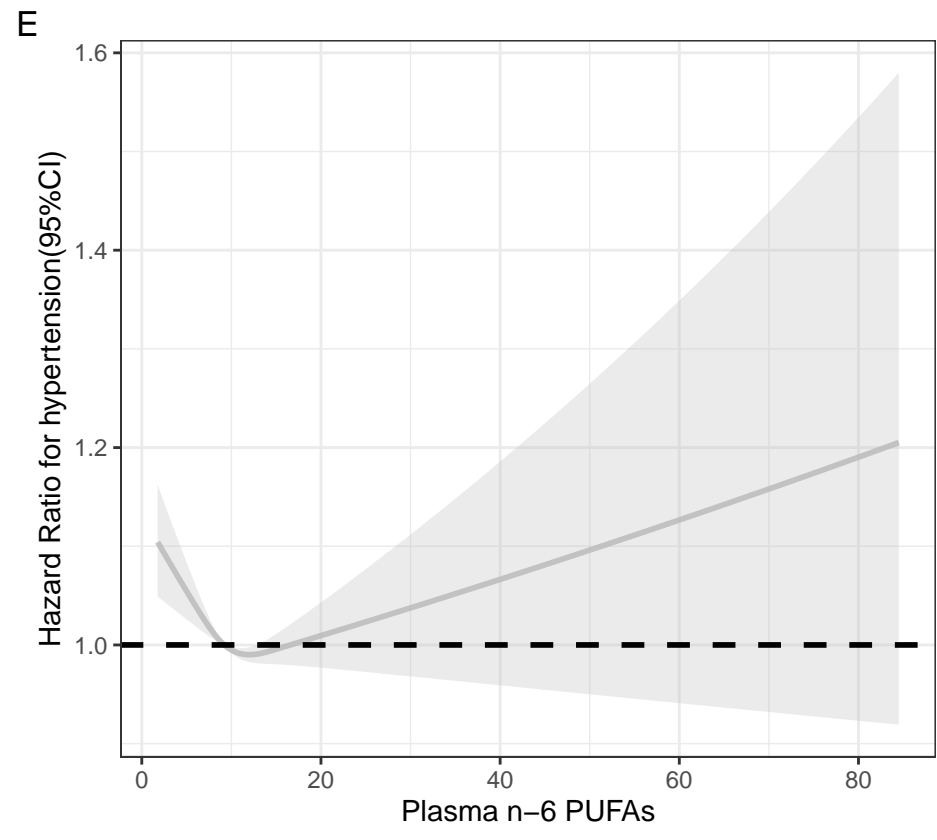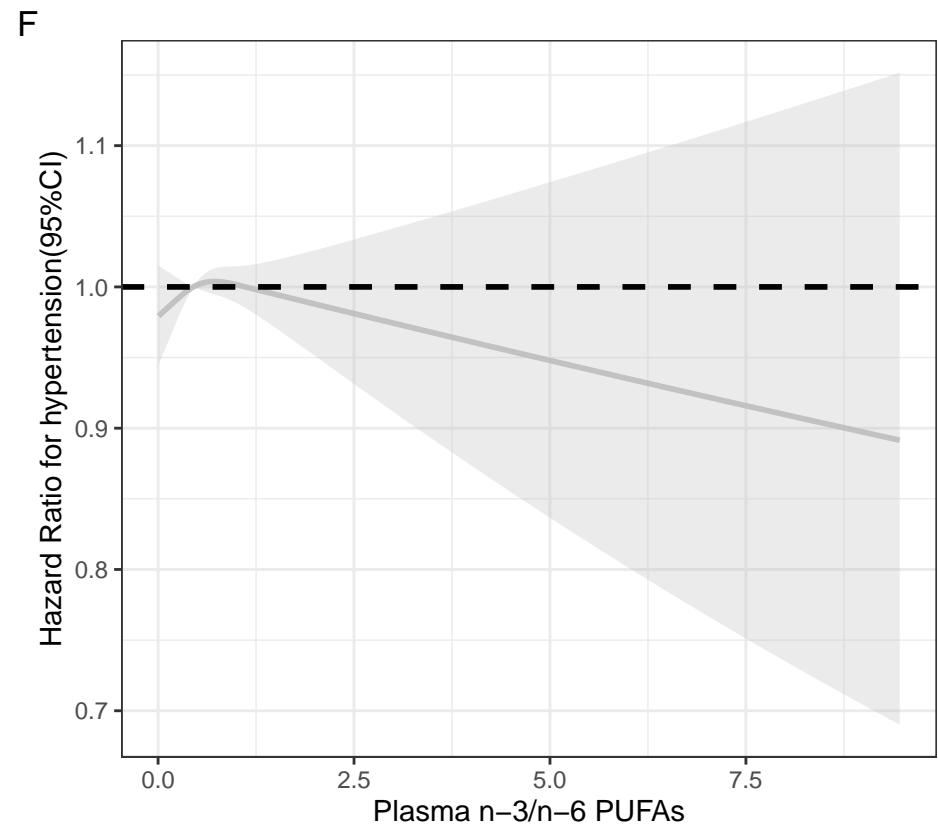

Supplement: SUPPLEMENTARY FIGURE S8 — Dose-response relationships between plasma FA and hypertension risk. HRs for hypertension associated with plasma PUFAs (A), MUFAs (B), SFAs (C), n-3 PUFAs (D), n-6 PUFAs (E), and n-3/n-6 ratio (F) were estimated by restricted cubic-spline regression using model 2 excluding participants with were any type of cardiovascular disease at baseline in the UK Biobank. [file Image_8.PDF]

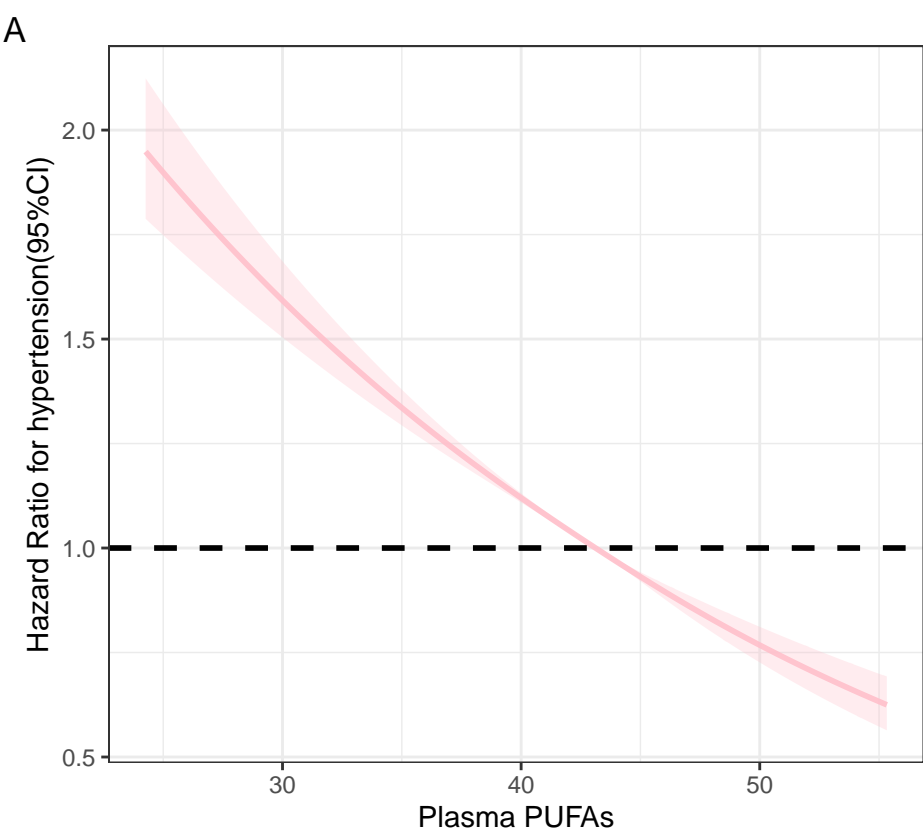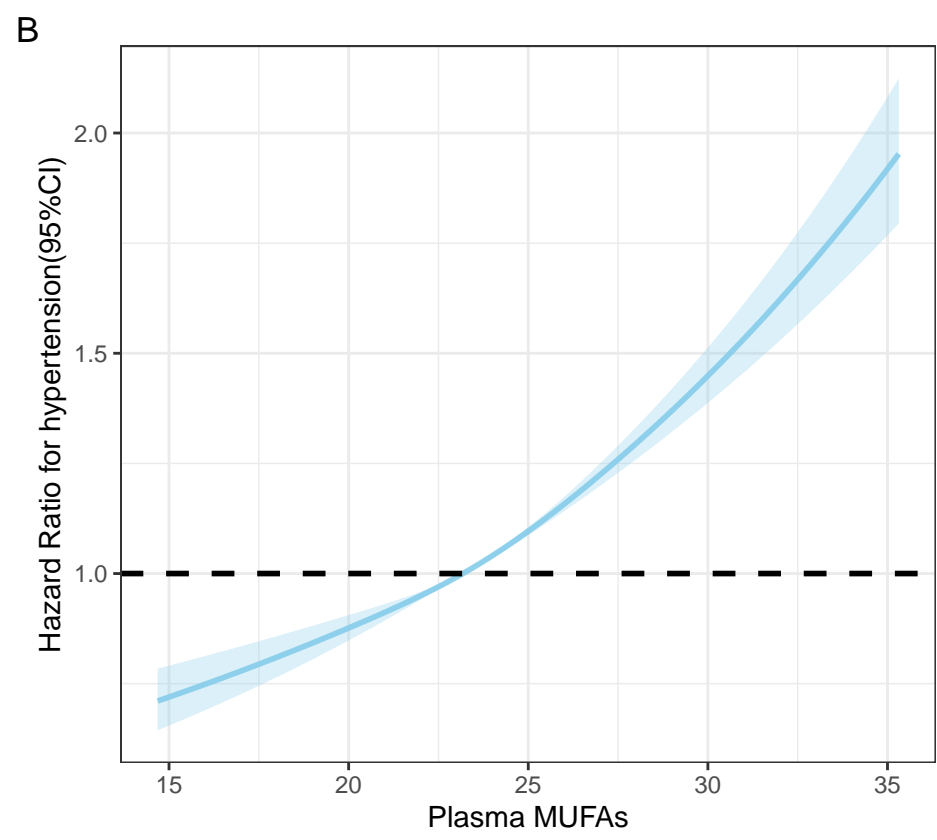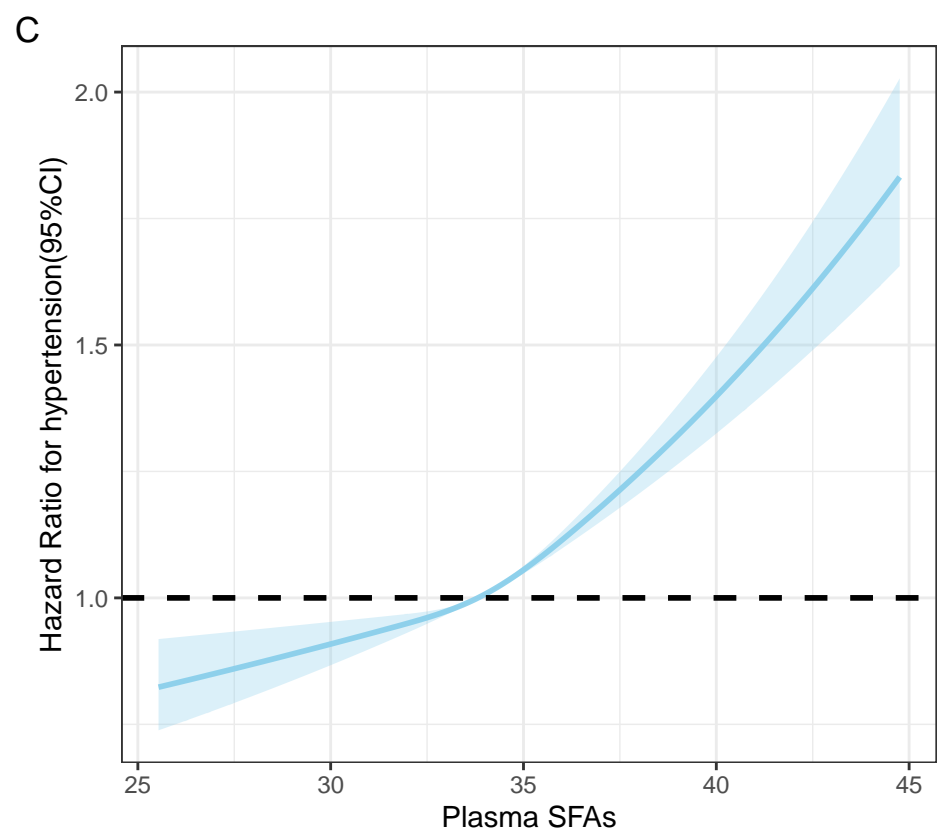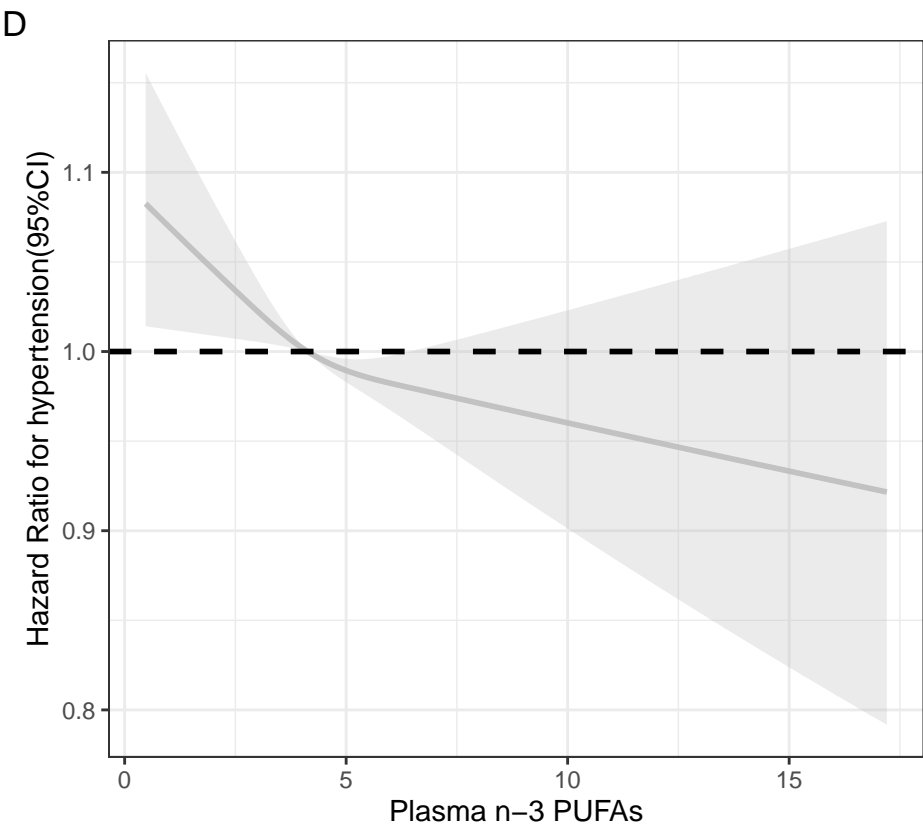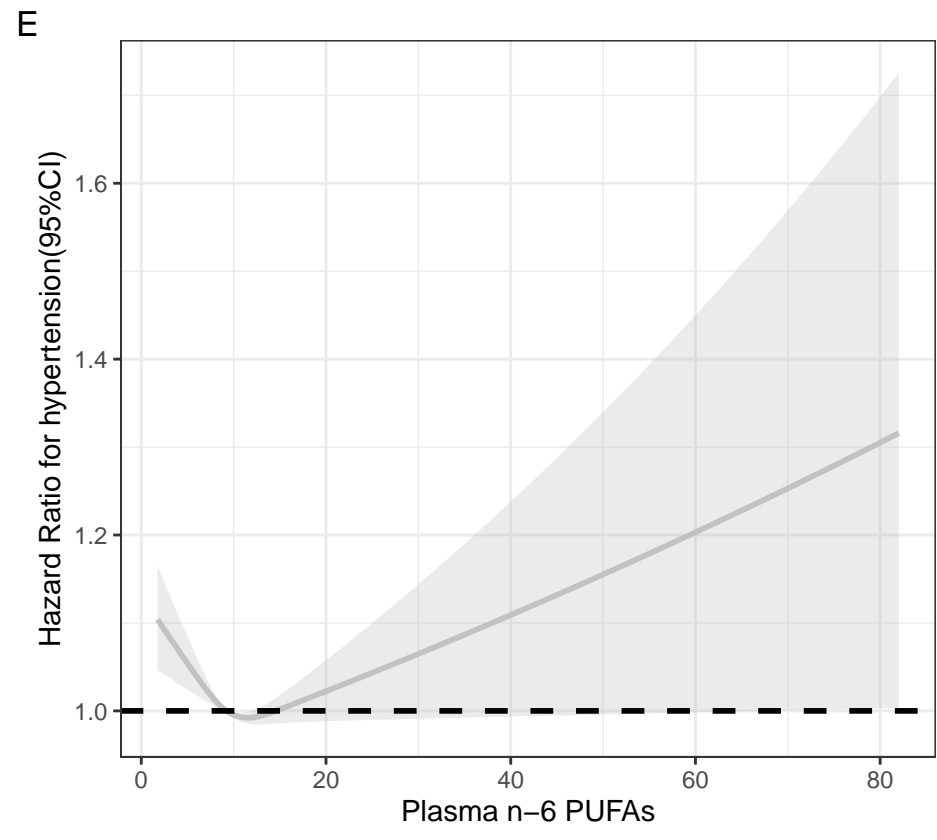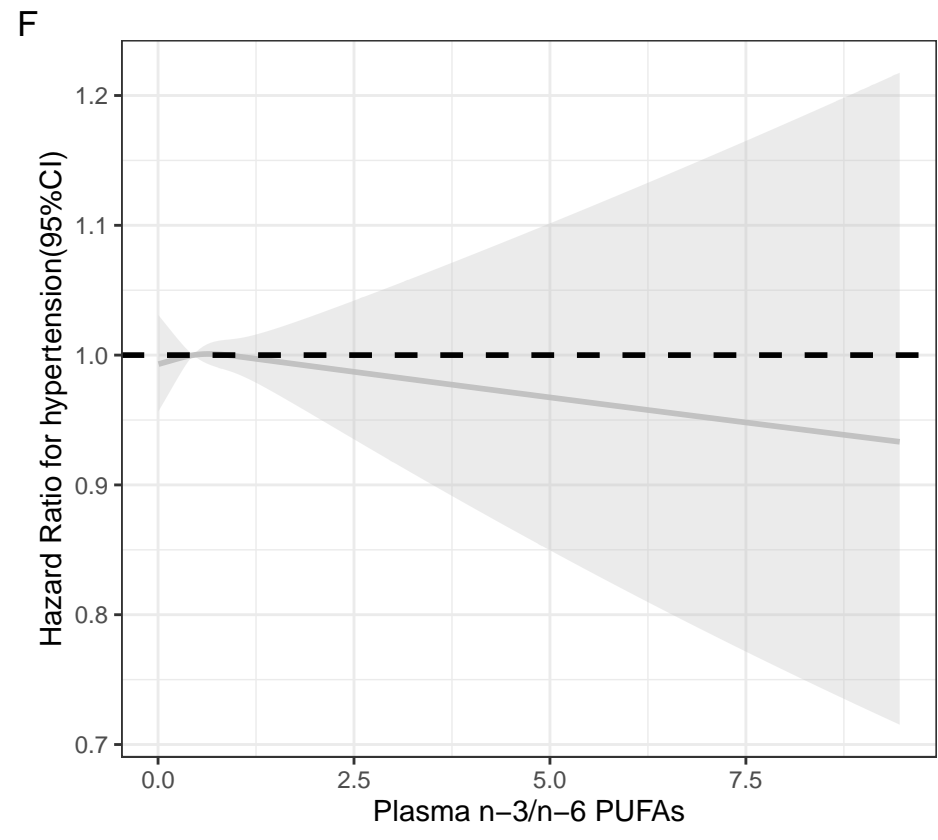

Supplement: SUPPLEMENTARY FIGURE S9 — Dose-response relationships between plasma FA and hypertension risk. HRs for hypertension associated with plasma PUFAs (A), MUFAs (B), SFAs (C), n-3 PUFAs (D), n-6 PUFAs (E), and n-3/n-6 ratio (F) were estimated by restricted cubic-spline regression using model 2 excluding participants with follow-up time of less than 2 years in the UK Biobank. [file Image_9.PDF]

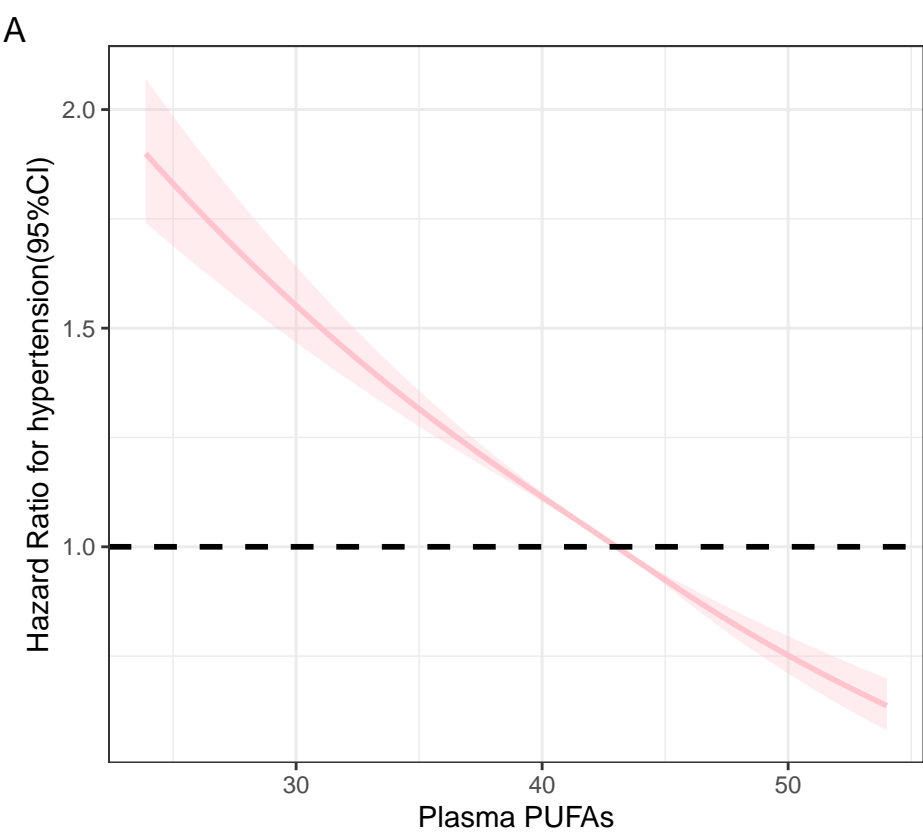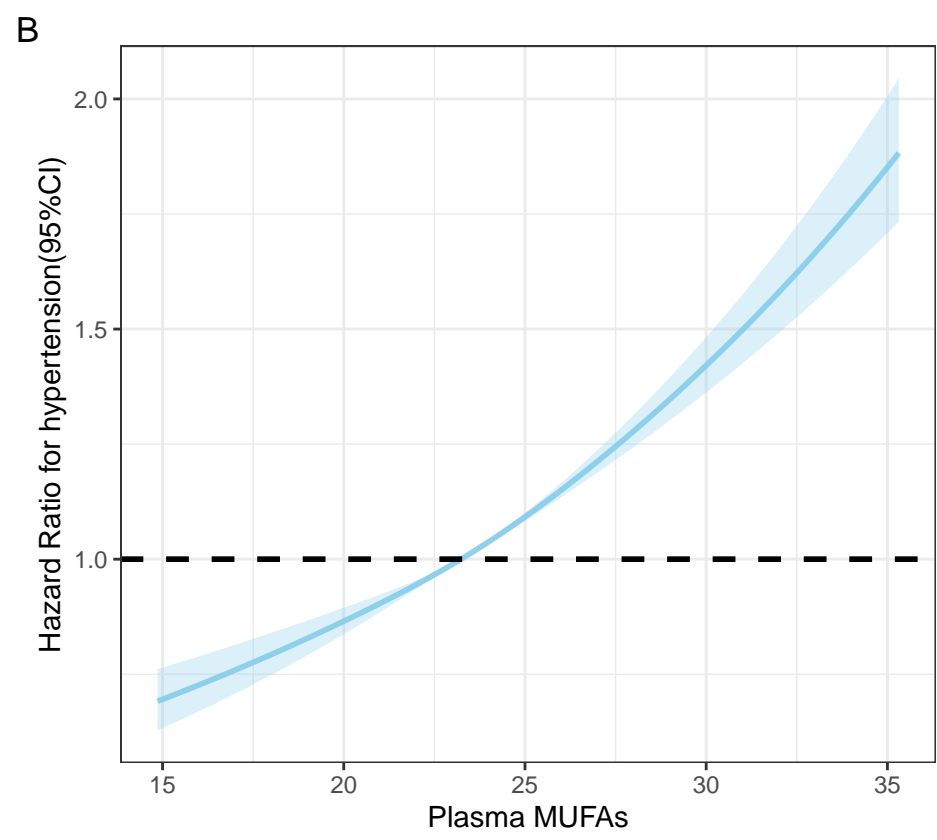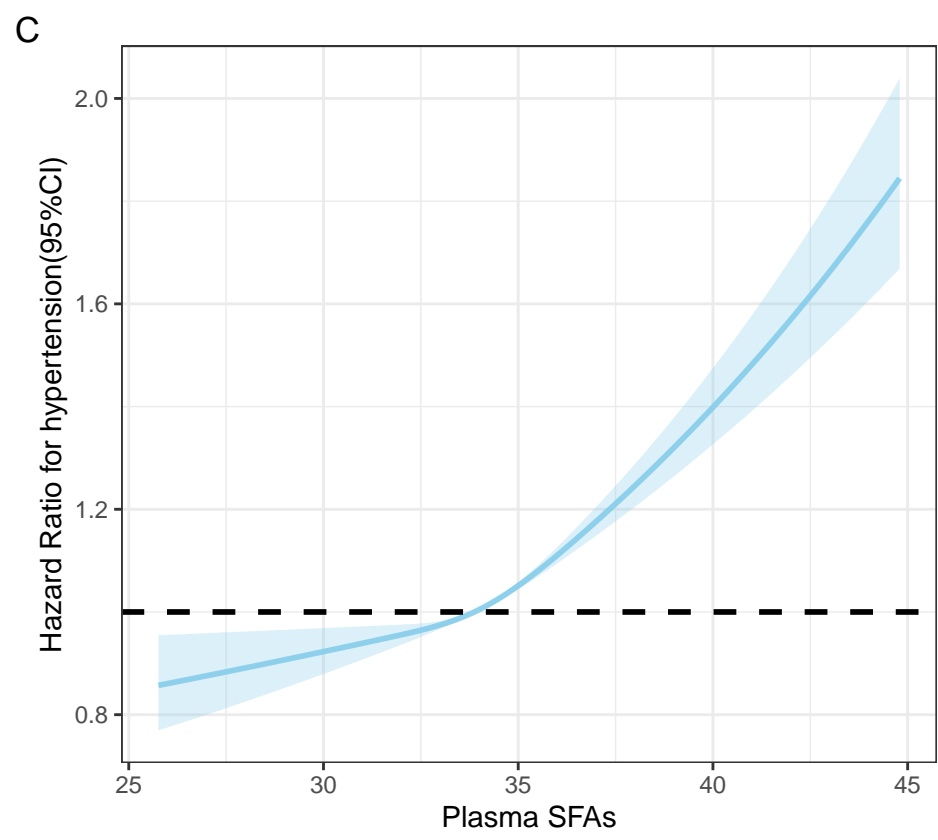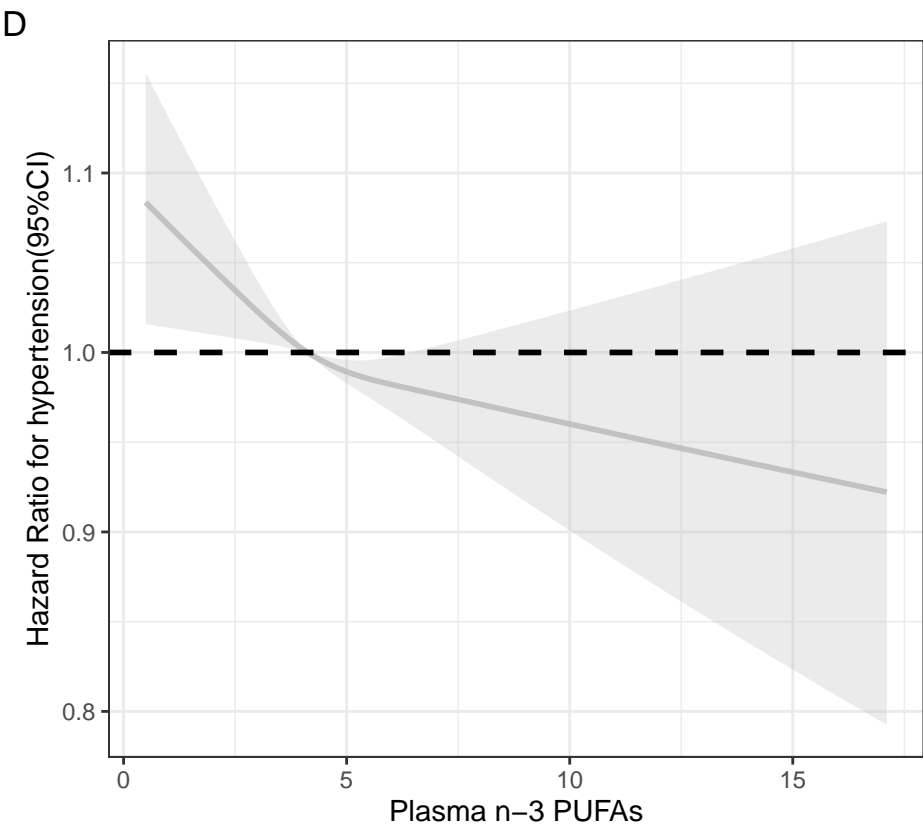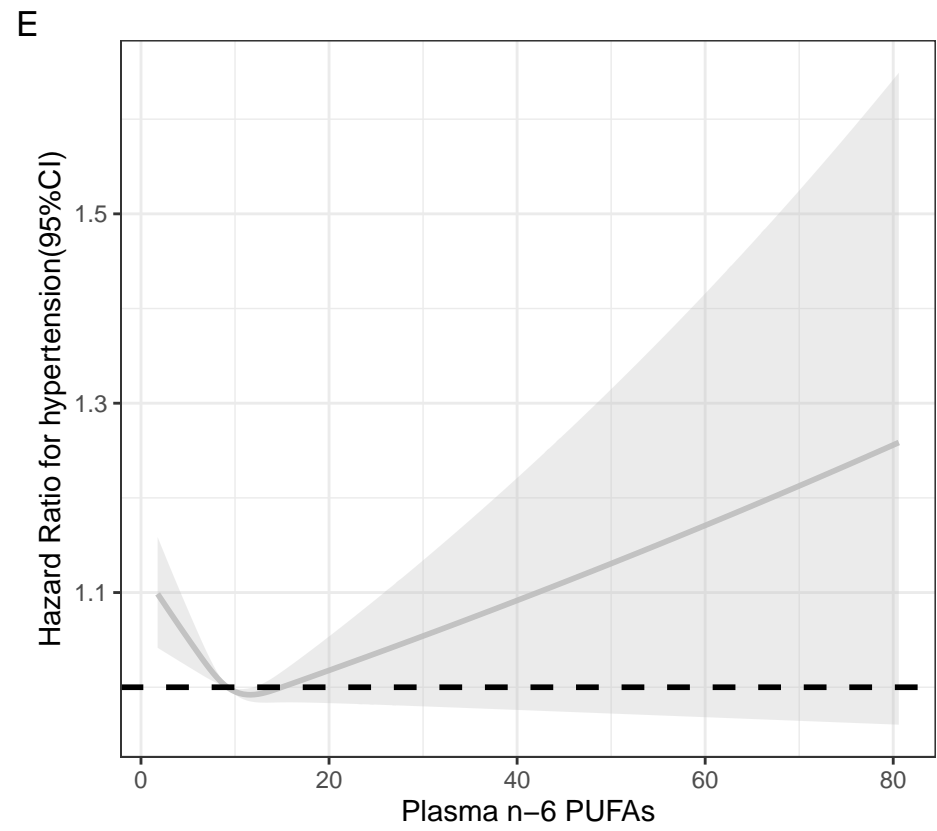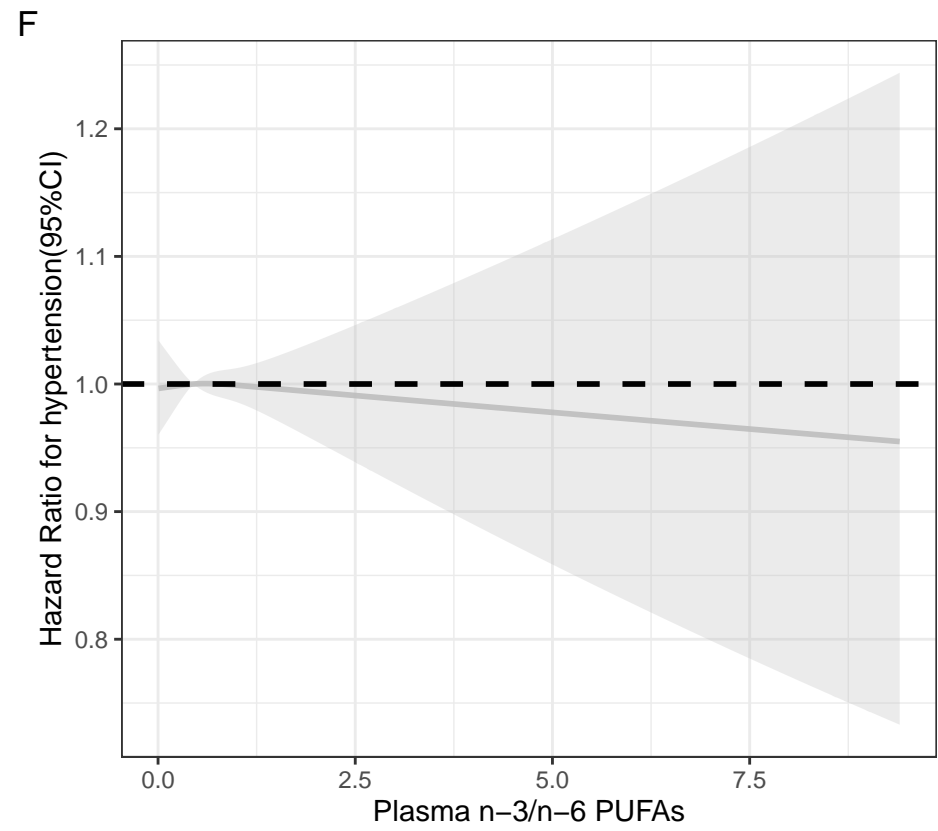

Supplement: SUPPLEMENTARY FIGURE S10 — Dose-response relationships between plasma FA and hypertension risk. HRs for hypertension associated with plasma PUFAs (A), MUFAs (B), SFAs (C), n-3 PUFAs (D), n-6 PUFAs (E), and n-3/n-6 ratio (F) were estimated by restricted cubic-spline regression using model 2 only race White participants in the UK Biobank. [file Image_10.PDF]

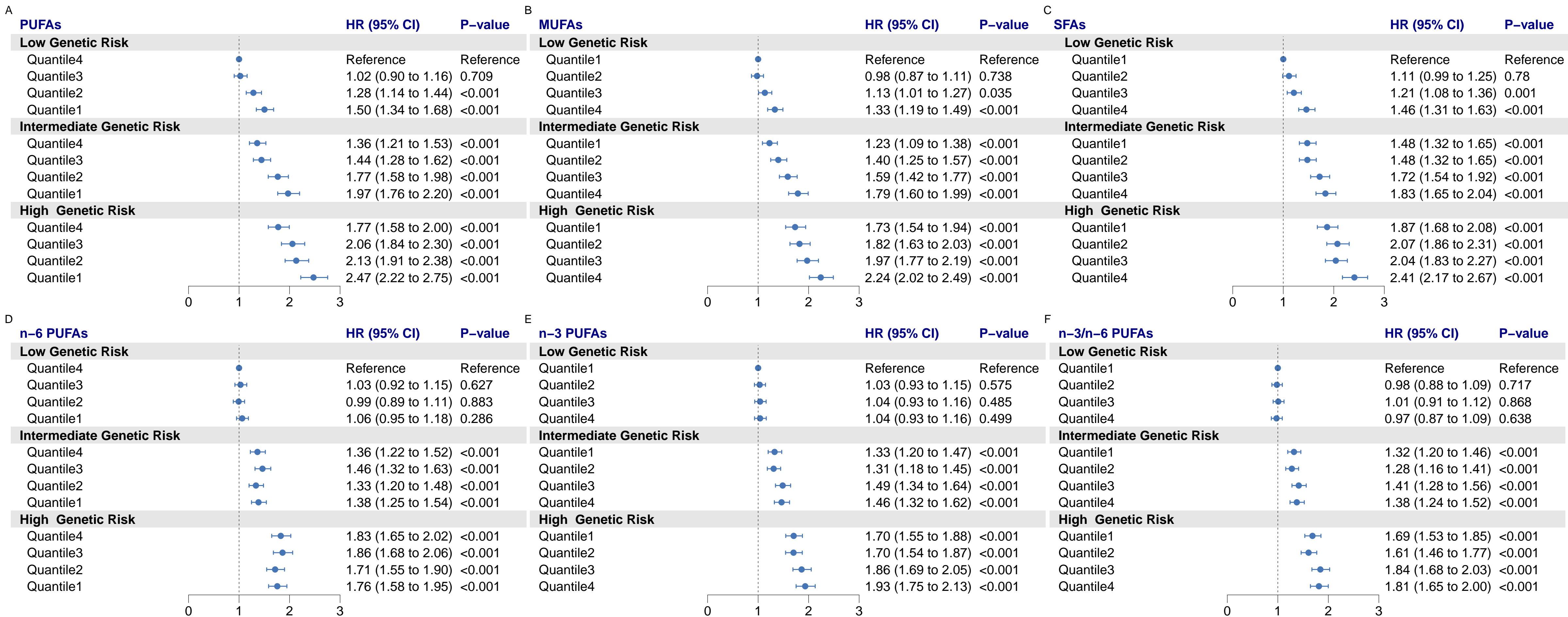

Supplement: SUPPLEMENTARY FIGURE S11 — Risk of incident hypertension according to concentration of plasma fatty acids and genetic risk categories. The HRs for hypertension according to PUFAs (A), MUFAs (B), SFAs (C), n-6 PUFAs (D), and polygenic risk score categories were estimated by using model 2 additionally adjusted stains plus the first 10 genetic principal components. [file Image_11.PDF]

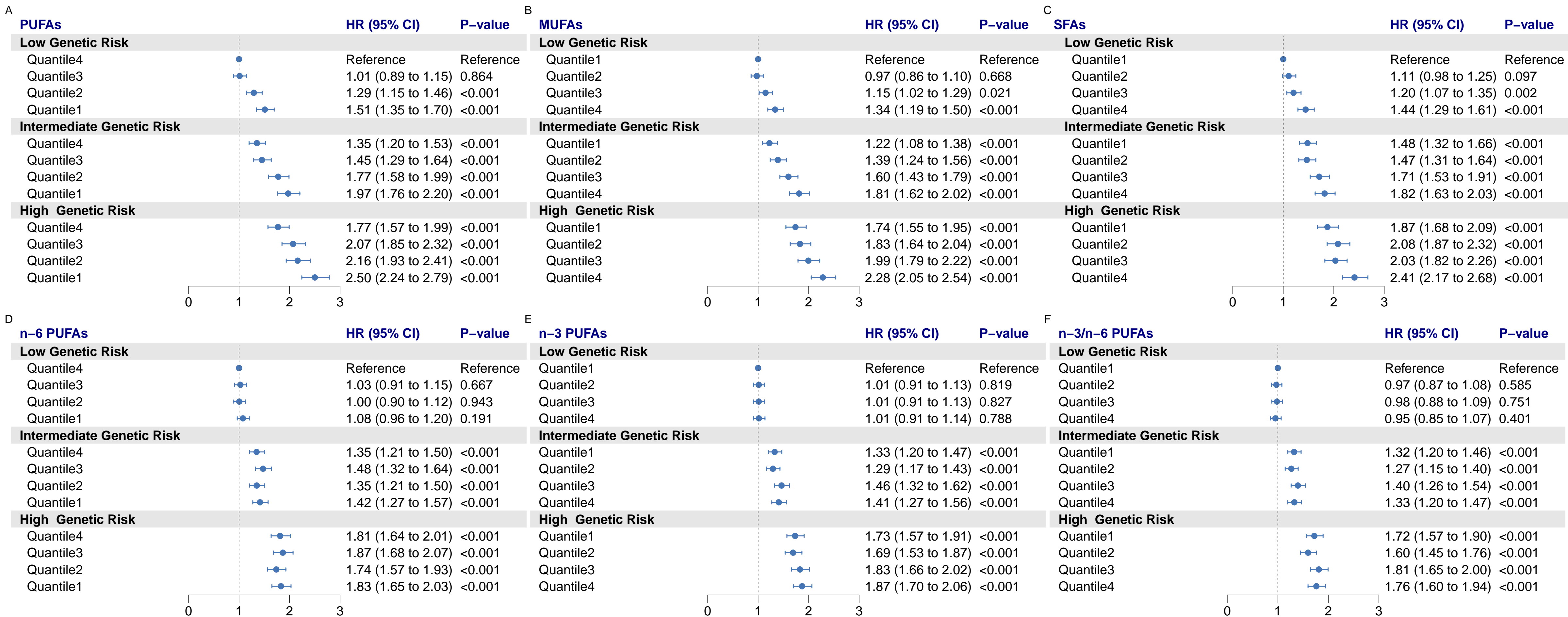

Supplement: SUPPLEMENTARY FIGURE S12 — Risk of incident hypertension according to concentration of plasma fatty acids and genetic risk categories. The HRs for hypertension according to PUFAs (A), MUFAs (B), SFAs (C), n-6 PUFAs (D), n-3 PUFAs (E), n-3/n-6 PUFAs (F), and polygenic risk score categories were estimated by using model 2 plus the first 10 genetic principal components excluding participants with were any type of cardiovascular disease at baseline in the UK Biobank. [file Image_12.PDF]

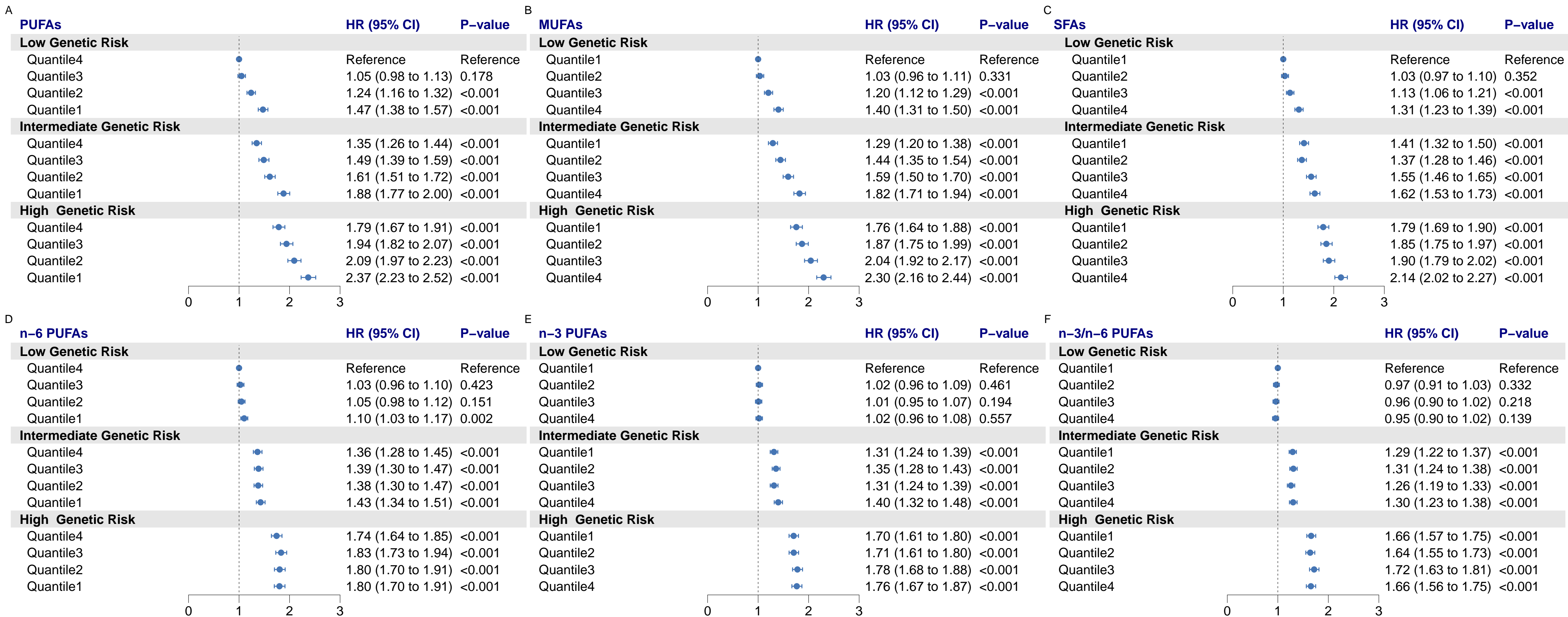

Supplement: SUPPLEMENTARY FIGURE S13 — Risk of incident hypertension according to concentration of plasma fatty acids and genetic risk categories. The HRs for hypertension according to PUFAs (A), MUFAs (B), SFAs (C), n-6 PUFAs (D), n-3 PUFAs (E), n-3/n-6 PUFAs (F), and polygenic risk score categories were estimated by using model 2 plus the first 10 genetic principal components excluding participants with follow-up time of less than 2 years in the UK Biobank. [file Image_13.PDF]

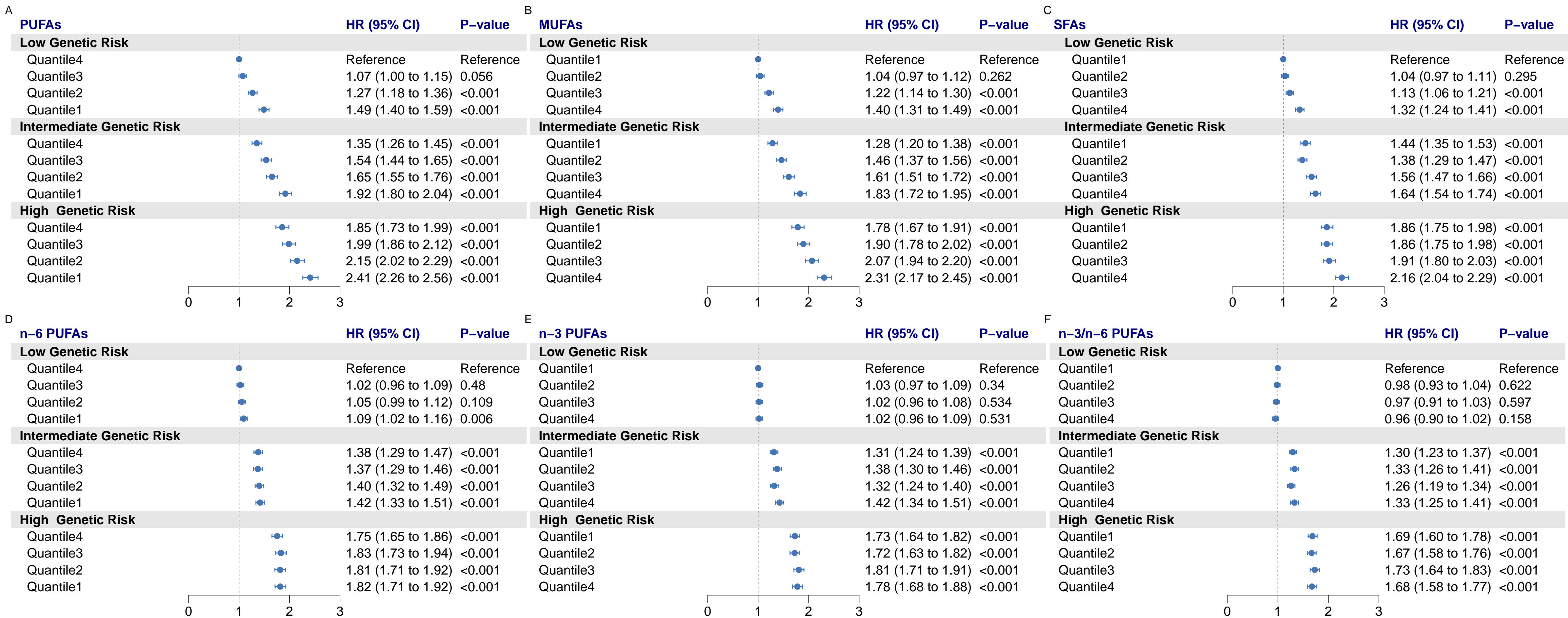

Supplement: SUPPLEMENTARY FIGURE S14 — Risk of incident hypertension according to concentration of plasma fatty acids and genetic risk categories. The HRs for hypertension according to PUFAs (A), MUFAs (B), SFAs (C), n-6 PUFAs (D), n-3 PUFAs (E), n-3/n-6 PUFAs (F), and polygenic risk score categories were estimated by using model 2 plus the first 10 genetic principal components only race White participants in the UK Biobank. [file Image_14.PDF]
