# Supplementary material for: Circulating fatty acids, genetic susceptibility and hypertension: a prospective cohort study
Source: Front Nutr. 2024 Oct 31;11:1454364. doi: 10.3389/fnut.2024.1454364 (PMC11562856; doi:10.3389/fnut.2024.1454364)
Supplement: Supplementary file 15 [file Table_1.docx]

**Circulating Fatty Acids, Genetic Risk and Hypertension: Nutrient-Gene Interaction Analysis**

Lingling Lu^1, *^, Xiaoli Gu^2,*^, Daheng Yang^3, #^, Bingjian Wang^4,#^, Guangfeng Long^3,#^

**Supplementary Materials Tables of Contents**

**Supplementary Table 1.** Components of more recent dietary recommendations for cardiovascular health#(1)

**Supplementary Table 2.** Clinical characteristics by quantile of MUFAs and SFAs (n= 195,819).

**Supplementary Table 3.** Stratified analyses for plasma fatty acids for hypertension risk.

**Supplementary Table 4.** Associations between hypertension risk and genetic factors according to deciles of PRS

**Supplementary Table 5.** Adjusted hazard ratios and 95% confidence interval for hypertension genetic risk score with the risk levels of incident hypertension.

**Supplementary Table 6.** Additionally adjusted stains hazard ratio and 95% confidence interval of hypertension by specific plasma FA exposure.

**Supplementary Table 7.** Adjusted hazard ratio and 95% confidence interval of hypertension by specific plasma FA exposure excluding participants with were any type of cardiovascular disease at baseline in the UK Biobank.

**Supplementary Table 8.** Adjusted hazard ratio and 95% confidence interval of hypertension by specific plasma FA exposure excluding participants with follow-up time of less than 2 years in the UK Biobank.

**Supplementary Table 9.** Adjusted hazard ratio and 95% confidence interval of hypertension by plasma FA exposure only race White participants in the UK Biobank.

**Supplementary Table 10.** RERI and AP for additive interaction between specific plasma fatty acids and genetic categories additionally adjusted stains.

**Supplementary Table 11.** RERI and AP for additive interaction between specific plasma fatty acids and genetic categories excluding participants with were any type of cardiovascular disease at baseline in the UK Biobank.

**Supplementary Table 12.** RERI and AP for additive interaction between specific plasma fatty acids and genetic categories excluding participants with follow-up time of less than 2 years in the UK Biobank.

**Supplementary Table 13.** RERI and AP for additive interaction between shift work exposure and genetic categories only race White participants in the UK Biobank.

**Supplementary Materials Figures of Contents**

**Supplementary Figure 1.** Risk of incident hypertension according to concentration of plasma fatty acids and genetic risk categories of female subgroup.

The HRs for hypertension according to PUFAs (A), MUFAs (B), SFAs (C), n-6 PUFAs (D), n-3 PUFAs (E), n-3/n-6 PUFAs (F), and polygenic risk score categories were estimated by using model 2 plus the first 10 genetic principal components.

**Supplementary Figure 2.** Risk of incident hypertension according to concentration of plasma fatty acids and genetic risk categories of male subgroup.

The HRs for hypertension according to PUFAs (A), MUFAs (B), SFAs (C), n-6 PUFAs (D), n-3 PUFAs (E), n-3/n-6 PUFAs (F), and polygenic risk score categories were estimated by using model 2 plus the first 10 genetic principal components.

**Supplementary Figure 3.** Risk of incident hypertension according to concentration of plasma fatty acids and genetic risk categories of <60 subgroup.

The HRs for hypertension according to PUFAs (A), MUFAs (B), SFAs (C), n-6 PUFAs (D), n-3 PUFAs (E), n-3/n-6 PUFAs (F), and polygenic risk score categories were estimated by using model 2 plus the first 10 genetic principal components.

**Supplementary Figure 4.** Risk of incident hypertension according to concentration of plasma fatty acids and genetic risk categories of ≥60 subgroup.

The HRs for hypertension according to PUFAs (A), MUFAs (B), SFAs (C), n-6 PUFAs (D), n-3 PUFAs (E), n-3/n-6 PUFAs (F), and polygenic risk score categories were estimated by using model 2 plus the first 10 genetic principal components.

**Supplementary Figure 5.** Risk of incident hypertension according to concentration of plasma fatty acids and genetic risk categories of normal-BMI subgroup.

The HRs for hypertension according to PUFAs (A), MUFAs (B), SFAs (C), n-6 PUFAs (D), n-3 PUFAs (E), n-3/n-6 PUFAs (F), and polygenic risk score categories were estimated by using model 2 plus the first 10 genetic principal components.

**Supplementary Figure 6.** Risk of incident hypertension according to concentration of plasma fatty acids and genetic risk categories of overweight-obesity BMI subgroup.

The HRs for hypertension according to PUFAs (A), MUFAs (B), SFAs (C), n-6 PUFAs (D), n-3 PUFAs (E), n-3/n-6 PUFAs (F), and polygenic risk score categories were estimated by using model 2 plus the first 10 genetic principal components.

**Supplementary Figure 7.** Dose-response relationships between plasma FA and hypertension risk.

HRs for hypertension associated with plasma PUFAs (A), MUFAs (B), SFAs (C), n-3 PUFAs (D), n-6 PUFAs (E), and n-3/n-6 ratio (F) were estimated by restricted cubic-spline regression using model 2 additionally adjusted stains.

**Supplementary Figure 8.** Dose-response relationships between plasma FA and hypertension risk.

HRs for hypertension associated with plasma PUFAs (A), MUFAs (B), SFAs (C), n-3 PUFAs (D), n-6 PUFAs (E), and n-3/n-6 ratio (F) were estimated by restricted cubic-spline regression using model 2 excluding participants with were any type of cardiovascular disease at baseline in the UK Biobank.

**Supplementary Figure 9.** Dose-response relationships between plasma FA and hypertension risk.

HRs for hypertension associated with plasma PUFAs (A), MUFAs (B), SFAs (C), n-3 PUFAs (D), n-6 PUFAs (E), and n-3/n-6 ratio (F) were estimated by restricted cubic-spline regression using model 2 excluding participants with follow-up time of less than 2 years in the UK Biobank.

**Supplementary Figure 10.** Dose-response relationships between plasma FA and hypertension risk.

HRs for hypertension associated with plasma PUFAs (A), MUFAs (B), SFAs (C), n-3 PUFAs (D), n-6 PUFAs (E), and n-3/n-6 ratio (F) were estimated by restricted cubic-spline regression using model 2 only race White participants in the UK Biobank.

**Supplementary Figure 11.** Risk of incident hypertension according to concentration of plasma fatty acids and genetic risk categories.

The HRs for hypertension according to PUFAs (A), MUFAs (B), SFAs (C), n-6 PUFAs (D), and polygenic risk score categories were estimated by using model 2 additionally adjusted stains plus the first 10 genetic principal components.

**Supplementary Figure 12.** Risk of incident hypertension according to concentration of plasma fatty acids and genetic risk categories.

The HRs for hypertension according to PUFAs (A), MUFAs (B), SFAs (C), n-6 PUFAs (D), n-3 PUFAs (E), n-3/n-6 PUFAs (F), and polygenic risk score categories were estimated by using model 2 plus the first 10 genetic principal components excluding participants with were any type of cardiovascular disease at baseline in the UK Biobank.

**Supplementary Figure 13.** Risk of incident hypertension according to concentration of plasma fatty acids and genetic risk categories.

The HRs for hypertension according to PUFAs (A), MUFAs (B), SFAs (C), n-6 PUFAs (D), n-3 PUFAs (E), n-3/n-6 PUFAs (F), and polygenic risk score categories were estimated by using model 2 plus the first 10 genetic principal components excluding participants with follow-up time of less than 2 years in the UK Biobank.

**Supplementary Figure 14.** Risk of incident hypertension according to concentration of plasma fatty acids and genetic risk categories.

The HRs for hypertension according to PUFAs (A), MUFAs (B), SFAs (C), n-6 PUFAs (D), n-3 PUFAs (E), n-3/n-6 PUFAs (F), and polygenic risk score categories were estimated by using model 2 plus the first 10 genetic principal components only race White participants in the UK Biobank.

**Supplementary table 1.** Components of more recent dietary recommendations for cardiovascular health#(1)

| Diet Component | Healthy Intake Goal | Field ID | Point* |
| --- | --- | --- | --- |
| Fruit | ≥3 servings/day | 1309 (pieces fresh fruit/day) 1319 (pieces dried fruit/day) | 1309 – 1 piece  1319 – 5 pieces |
| Vegetable | ≥3 servings/day | 1289 (tablespoons cooked vegetables/day) 1299 (salad/raw vegetables/day) | 3 heaped tablespoons |
| Whole grains | ≥3servings/day | 1438, 1448 (wholemeal/wholegrain bread slices/week)  1458, 1468 (bran/oat/muesli cereal bowls/week) | 1438/1448 – 1 slice/day 1458/1468 – 1 bowl/day |
| (Shell)fish | ≥2 servings/week | 1329 (oily fish/week)  1339 (non-oily fish/week) | Once/week |
| Dairy | ≥2 servings/day | 1408 (cheese/week) 1418 (milk type) | 1408 – 1 piece/day  1418 – 1 glass/day if consumption of any type of milk |
| Vegetable oils | ≥2servings/day | 1428 (Flora Pro-Active/Benecol spread)  2654 (Flora Pro-Active/Benecol, soft margarine -, olive oil based -, polyunsaturated/sunflower oil  based -, other low/reduced fat spread) 1438 (bread slices/week) | 1 serving/day if in combination with eating at least 2 slices of bread (ID 1438) |
| Refined grains | ≤servings/day | 1438, 1448 (white, brown, other bread slices/week) 1458, 1468 (biscuit, other cereals/week) | 1438/1448 – 1 slice/day 1458/1468 – 1 bowl/day |
| Processed meats | ≤1serving/week | 1349 (processed meat/week or daily) 3680 (age when lastate meat) | 1349 – 1 piece/day  3680 – 0 pieces/day if indicated having never eaten meat |
| Unprocessed meats | ≤2servings/week | 1359 (poultry/week or day) 1369 (beef/week or day)  1379 (lamb or mutton/week or day) 1389 (pork/week or day)  3680 (age when lastate meat) | 1359-1389 – once/week  3680 – 0 pieces/day if indicated having never eaten meat |
| Sugar-sweetened beverages | No-consumption | 6144 (never consumes drinks containing sugar) | Only 0 servings were possible here. |

* 1 point for individuals meet the healthy intake goal of dietary recommendation and summarize the total score.

# The healthy dietary was defined as the total score more than 5 indicating meeting at least half of the dietary recommendation at least 5 items.

**Supplementary Table 2.** Clinical characteristics by quantile of MUFAs and SFAs (n= 195,819).

|  | **MUFAs** | | | | **SFAs** | | | |
| --- | --- | --- | --- | --- | --- | --- | --- | --- |
|  | **Quantile 1** | **Quantile 2** | **Quantile 3** | **Quantile 4** | **Quantile 1** | **Quantile 2** | **Quantile 3** | **Quantile 4** |
|  | (N=48817) | (N=48818) | (N=48818) | (N=48797) | (N=48851) | (N=48771) | (N=48819) | (N=48809) |
| **Age (years), mean (SD)** | 54.6 (8.20) | 55.5 (8.13) | 55.9 (8.12) | 55.7 (8.12) | 54.5 (8.26) | 55.2 (8.15) | 55.7 (8.11) | 56.2 (8.00) |
| **Sex, %** |  |  |  |  |  |  |  |  |
| Female | 34532 (70.7%) | 30369 (62.2%) | 26164 (53.6%) | 18757 (38.4%) | 26286 (53.8%) | 29462 (60.4%) | 29428 (60.3%) | 24646 (50.5%) |
| Male | 14285 (29.3%) | 18449 (37.8%) | 22654 (46.4%) | 30040 (61.6%) | 22565 (46.2%) | 19309 (39.6%) | 19391 (39.7%) | 24163 (49.5%) |
| **Race, %** |  |  |  |  |  |  |  |  |
| White | 45276 (92.7%) | 46218 (94.7%) | 46577 (95.4%) | 47059 (96.4%) | 43876 (89.8%) | 46555 (95.5%) | 47237 (96.8%) | 47462 (97.2%) |
| Asian or Asian British | 699 (1.4%) | 972 (2.0%) | 982 (2.0%) | 720 (1.5%) | 1895 (3.9%) | 646 (1.3%) | 412 (0.8%) | 420 (0.9%) |
| Black or Black British | 1514 (3.1%) | 499 (1.0%) | 226 (0.5%) | 128 (0.3%) | 1176 (2.4%) | 579 (1.2%) | 379 (0.8%) | 233 (0.5%) |
| Chinese | 198 (0.4%) | 187 (0.4%) | 143 (0.3%) | 117 (0.2%) | 410 (0.8%) | 131 (0.3%) | 66 (0.1%) | 38 (0.1%) |
| Mixed | 374 (0.8%) | 302 (0.6%) | 253 (0.5%) | 227 (0.5%) | 346 (0.7%) | 288 (0.6%) | 278 (0.6%) | 244 (0.5%) |
| Other ethnic group | 532 (1.1%) | 422 (0.9%) | 408 (0.8%) | 314 (0.6%) | 834 (1.7%) | 353 (0.7%) | 272 (0.6%) | 217 (0.4%) |
| Missing value | 224 (0.5%) | 218 (0.4%) | 229 (0.5%) | 232 (0.5%) | 314 (0.6%) | 219 (0.4%) | 175 (0.4%) | 195 (0.4%) |
| **BMI, %** |  |  |  |  |  |  |  |  |
| Normal (<25 kg/m2) | 29239 (59.9%) | 21580 (44.2%) | 14930 (30.6%) | 8707 (17.8%) | 21369 (43.7%) | 20005 (41.0%) | 18849 (38.6%) | 14233 (29.2%) |
| Overweight (25 to 29.9 kg/m2) | 16097 (33.0%) | 20609 (42.2%) | 23018 (47.2%) | 23555 (48.3%) | 20329 (41.6%) | 20573 (42.2%) | 20656 (42.3%) | 21721 (44.5%) |
| Obesity (≥30 kg/m2) | 3331 (6.8%) | 6503 (13.3%) | 10742 (22.0%) | 16329 (33.5%) | 6968 (14.3%) | 8060 (16.5%) | 9173 (18.8%) | 12704 (26.0%) |
| Missing value | 150 (0.3%) | 126 (0.3%) | 128 (0.3%) | 206 (0.4%) | 185 (0.4%) | 133 (0.3%) | 141 (0.3%) | 151 (0.3%) |
| **Smoke status, %** |  |  |  |  |  |  |  |  |
| Never | 30684 (62.9%) | 28842 (59.1%) | 27004 (55.3%) | 23077 (47.3%) | 30073 (61.6%) | 28585 (58.6%) | 27242 (55.8%) | 23707 (48.6%) |
| Previous | 14931 (30.6%) | 15451 (31.7%) | 15961 (32.7%) | 17113 (35.1%) | 14424 (29.5%) | 15278 (31.3%) | 15980 (32.7%) | 17774 (36.4%) |
| Current | 3002 (6.1%) | 4312 (8.8%) | 5626 (11.5%) | 8325 (17.1%) | 4111 (8.4%) | 4683 (9.6%) | 5390 (11.0%) | 7081 (14.5%) |
| Missing value | 200 (0.4%) | 213 (0.4%) | 227 (0.5%) | 282 (0.6%) | 243 (0.5%) | 225 (0.5%) | 207 (0.4%) | 247 (0.5%) |
| **Alcohol Consumption, %** |  |  |  |  |  |  |  |  |
| Daily or almost daily | 10943 (22.4%) | 9563 (19.6%) | 8909 (18.2%) | 9033 (18.5%) | 5506 (11.3%) | 8200 (16.8%) | 10793 (22.1%) | 13949 (28.6%) |
| Three or four times a week | 12908 (26.4%) | 11905 (24.4%) | 11237 (23.0%) | 10641 (21.8%) | 9986 (20.4%) | 11855 (24.3%) | 12646 (25.9%) | 12204 (25.0%) |
| Once or twice a week | 12386 (25.4%) | 13148 (26.9%) | 13407 (27.5%) | 13166 (27.0%) | 14174 (29.0%) | 13997 (28.7%) | 12775 (26.2%) | 11161 (22.9%) |
| One to three times a month | 4953 (10.1%) | 5450 (11.2%) | 5739 (11.8%) | 6046 (12.4%) | 6995 (14.3%) | 5832 (12.0%) | 5027 (10.3%) | 4334 (8.9%) |
| Special occasions only | 4409 (9.0%) | 5132 (10.5%) | 5542 (11.4%) | 5908 (12.1%) | 6715 (13.7%) | 5432 (11.1%) | 4604 (9.4%) | 4240 (8.7%) |
| Never | 3107 (6.4%) | 3536 (7.2%) | 3881 (8.0%) | 3891 (8.0%) | 5336 (10.9%) | 3370 (6.9%) | 2898 (5.9%) | 2811 (5.8%) |
| Missing value | 111 (0.2%) | 84 (0.2%) | 103 (0.2%) | 112 (0.2%) | 139 (0.3%) | 85 (0.2%) | 76 (0.2%) | 110 (0.2%) |
| **Townsend deprivation index** | -1.63 (2.96) | -1.58 (2.94) | -1.45 (3.00) | -1.15 (3.14) | -1.27 (3.13) | -1.55 (2.96) | -1.56 (2.95) | -1.43 (3.02) |
| **Family history, %** |  |  |  |  |  |  |  |  |
| CVD | 35158 (72.0%) | 35557 (72.8%) | 35570 (72.9%) | 35416 (72.6%) | 35642 (73.0%) | 35494 (72.8%) | 35240 (72.2%) | 35325 (72.4%) |
| Other | 13097 (26.8%) | 12637 (25.9%) | 12641 (25.9%) | 12743 (26.1%) | 12629 (25.9%) | 12670 (26.0%) | 12977 (26.6%) | 12842 (26.3%) |
| No Disease | 562 (1.2%) | 624 (1.3%) | 607 (1.2%) | 638 (1.3%) | 580 (1.2%) | 607 (1.2%) | 602 (1.2%) | 642 (1.3%) |
| **Diet Score** |  |  |  |  |  |  |  |  |
| Unhealthy | 30885 (63.3%) | 32177 (65.9%) | 33381 (68.4%) | 35419 (72.6%) | 30301 (62.0%) | 32269 (66.2%) | 33788 (69.2%) | 35504 (72.7%) |
| Healthy | 15097 (30.9%) | 13988 (28.7%) | 12747 (26.1%) | 10535 (21.6%) | 15377 (31.5%) | 13915 (28.5%) | 12397 (25.4%) | 10678 (21.9%) |
| Missing | 2835 (5.8%) | 2653 (5.4%) | 2690 (5.5%) | 2843 (5.8%) | 3173 (6.5%) | 2587 (5.3%) | 2634 (5.4%) | 2627 (5.4%) |
| **Physical Activity, %** |  |  |  |  |  |  |  |  |
| No | 5648 (11.6%) | 6088 (12.5%) | 6971 (14.3%) | 8060 (16.5%) | 6605 (13.5%) | 6436 (13.2%) | 6496 (13.3%) | 7230 (14.8%) |
| Yes | 33516 (68.7%) | 32354 (66.3%) | 30888 (63.3%) | 29299 (60.0%) | 32100 (65.7%) | 31802 (65.2%) | 31464 (64.5%) | 30691 (62.9%) |
| Unkown | 9653 (19.8%) | 10376 (21.3%) | 10959 (22.4%) | 11438 (23.4%) | 10146 (20.8%) | 10533 (21.6%) | 10859 (22.2%) | 10888 (22.3%) |

**Supplement Table 3.** Stratified analyses for plasma fatty acids for hypertension risk.

|  | PUFA | MUFA | SFA | n-6 PUFA | n-3 PUFA | n-3/n-6 PUFA |
| --- | --- | --- | --- | --- | --- | --- |
|  | HR (95%CI) | HR (95%CI) | HR (95%CI) | HR (95%CI) | HR (95%CI) | HR (95%CI) |
| Gender |  |  |  |  |  |  |
| Male | 0.901 (0.889, 0.914) | 1.117 (1.100, 1.134) | 1.062 (1.048, 1.077) | 0.985 (0.971, 1.000) | 0.994 (0.979, 1.010) | 1.021 (0.980, 1.064) |
| Female | 0.845 (0.830, 0.860) | 1.160 (1.139, 1.181) | 1.122 (1.103, 1.141) | 1.003 (0.985, 1.021) | 0.973 (0.957, 0.989) | 1.000 (0.953, 1.049) |
| Age |  |  |  |  |  |  |
| ≥60 | 0.898 (0.885, 0.911) | 1.125 (1.108, 1.142) | 1.054 (1.038, 1.069) | 1.016 (1.000, 1.032) | 0.969 (0.955, 0.983) | 0.990 (0.947, 1.034) |
| <60 | 0.812 (0.799, 0.826) | 1.230 (1.209, 1.251) | 1.136 (1.117, 1.154) | 1.013 (0.997, 1.029) | 0.936 (0.919, 0.954) | 0.997 (0.954, 1.042) |
| BMI |  |  |  |  |  |  |
| Normal | 0.834 (0.814, 0.855) | 1.183 (1.154, 1.212) | 1.097 (1.072, 1.123) | 1.030 (1.009, 1.052) | 0.957 (0.938, 0.977) | 0.968 (0.908, 1.032) |
| Overweight | 0.857 (0.844, 0.871) | 1.182 (1.163, 1.202) | 1.081 (1.064, 1.098) | 0.994 (0.977, 1.012) | 0.971 (0.955, 0.987) | 1.031 (0.984, 1.082) |
| Obesity | 0.881 (0.864, 0.899) | 1.136 (1.113, 1.160) | 1.090 (1.070, 1.111) | 1.026 (1.005, 1.046) | 0.938 (0.917, 0.960) | 0.949 (0.899, 1.002) |
| Physical activity |  |  |  |  |  |  |
| No | 0.857 (0.833, 0.881) | 1.170 (1.136, 1.205) | 1.099 (1.068, 1.130) | 1.013 (0.983, 1.044) | 0.951 (0.922, 0.981) | 0.968 (0.892, 1.051) |
| Yes | 0.866 (0.854, 0.879) | 1.160 (1.143, 1.177) | 1.081 (1.066, 1.096) | 1.013 (0.998, 1.028) | 0.964 (0.950, 0.978) | 1.000 (0.960, 1.041) |
| TDI |  |  |  |  |  |  |
| Low | 0.864 (0.850, 0.879) | 1.165 (1.145, 1.185) | 1.081 (1.064, 1.099) | 1.016 (0.998, 1.034) | 0.966 (0.950, 0.982) | 1.016 (0.969, 1.066) |
| High | 0.852 (0.840, 0.865) | 1.178 (1.161, 1.197) | 1.094 (1.079, 1.111) | 1.013 (0.999, 1.028) | 0.951 (0.937, 0.966) | 0.969 (0.930, 1.010) |
| Smoke status |  |  |  |  |  |  |
| Never | 0.856 (0.842, 0.870) | 1.178 (1.158, 1.199) | 1.085 (1.067, 1.102) | 1.025 (1.009, 1.042) | 0.953 (0.938, 0.968) | 0.997 (0.953, 1.044) |
| Previous | 0.844 (0.820, 0.868) | 1.169 (1.136, 1.203) | 1.125 (1.093, 1.158) | 1.017 (0.991, 1.043) | 0.942 (0.909, 0.976) | 0.980 (0.908, 1.057) |
| Current | 0.866 (0.851, 0.881) | 1.166 (1.146, 1.188) | 1.079 (1.060, 1.097) | 0.997 (0.978, 1.017) | 0.969 (0.952, 0.987) | 0.990 (0.940, 1.043) |
| Alcohol consumption$ |  |  |  |  |  |  |
| Current | 0.857 (0.838, 0.877) | 1.161 (1.134, 1.189) | 1.099 (1.074, 1.125) | 1.013 (0.988, 1.039) | 0.965 (0.943, 0.988) | 0.957 (0.894, 1.023) |
| Sometimes | 0.876 (0.848, 0.905) | 1.186 (1.146, 1.228) | 1.043 (1.011, 1.077) | 1.019 (0.989, 1.051) | 0.972 (0.941, 1.005) | 1.041 (0.953, 1.137) |
| Never | 0.890 (0.859, 0.922) | 1.114 (1.072, 1.159) | 1.086 (1.051, 1.123) | 0.979 (0.949, 1.009) | 1.001 (0.967, 1.037) | 1.074 (0.976, 1.181) |

HR, hazard ratio. CI, confidence interval.

* per 1-SD; $ Daily or almost daily, three or four times a week and once or twice a week as current, one to three times a month and special occasions only as sometimes;

**Supplement Table 4.** Associations between hypertension risk and genetic factors according to deciles of PRS

| PRS | Model 1 | | Model 2 | |
| --- | --- | --- | --- | --- |
|  | HR (95% CI) | *P* value | HR (95% CI) | *P* value |
| Quintile 1 | Reference | Reference | Reference | Reference |
| Quintile 2 | 1.221 (1.155, 1.291) | < 0.001 | 1.240 (1.119, 1.374) | < 0.001 |
| Quintile 3 | 1.307 (1.237, 1.381) | < 0.001 | 1.356 (1.226, 1.500) | < 0.001 |
| Quintile 4 | 1.410 (1.335, 1.489) | < 0.001 | 1.469 (1.330, 1.623) | < 0.001 |
| Quintile 5 | 1.571 (1.489, 1.657) | < 0.001 | 1.626 (1.475, 1.792) | < 0.001 |
| Quintile 6 | 1.684 (1.597, 1.775) | < 0.001 | 1.766 (1.605, 1.944) | < 0.001 |
| Quintile 7 | 1.765 (1.675, 1.861) | < 0.001 | 1.817 (1.651, 2.001) | < 0.001 |
| Quintile 8 | 1.884 (1.789, 1.985) | < 0.001 | 1.841 (1.673, 2.027) | < 0.001 |
| Quintile 9 | 2.078 (1.974, 2.188) | < 0.001 | 2.138 (1.947, 2.349) | < 0.001 |
| Quintile 10 | 2.474 (2.352, 2.601) | < 0.001 | 2.600 (2.372, 2.851) | < 0.001 |
| *P* trend |  | < 0.001 |  | < 0.001 |

HR, hazard ratio. CI, confidence interval.

Model 1, adjusted for age and gender(male/female), genotyping batch, and the first 4 genetic principal components.

Model 2, model 1 plus race(White/Asian /Black/other/missing), Townsend deprivation index (TDI), healthy diet (unhealthy/healthy/missing), physical activity(yes/no/unknown), body mass index(BMI) (<25 kg/m2, 25 to 29.9 kg/m2, ≥30 kg/m2, missing), alcohol consumption (daily or almost daily/three or four times a week/once or twice a week/one to three times a month/special occasions only/never/missing), smoking status (never/before/current/missing), diabetes (yes/no/missing), family history of disease (CVD/other diseases/missing), and the first 10 genetic principal components.

**Supplement Table 5.** Adjusted hazard ratios and 95% confidence interval for hypertension genetic risk score with the risk levels of incident hypertension.

|  | Low | Intermediate | High | HR (95% CI) for the  continuous variable | P for trend |
| --- | --- | --- | --- | --- | --- |
| Model 1 | Reference | 1.334 (1.297, 1.372) | 1.751 (1.705, 1.798) | 1.319 (1.304, 1.334) | < 0.001 |
| Model 2 | Reference | 1.356 (1.289, 1.427) | 1.749 (1.665, 1.837) | 1.329 (1.301, 1.358) | < 0.001 |

HR, hazard ratio. CI, confidence interval.

Model 1, adjusted for age and gender(male/female), genotyping batch, and the first 4 genetic principal components.

Model 2, model 1 plus race(White/Asian /Black/other/missing), Townsend deprivation index (TDI), healthy diet (unhealthy/healthy/missing), physical activity(Yes/no/unknown), body mass index(BMI) (<25 kg/m2, 25 to 29.9 kg/m2, ≥30 kg/m2, missing), alcohol consumption (daily or almost daily/three or four times a week/once or twice a week/one to three times a month/special occasions only/never/missing), smoking status (never/before/current/missing), diabetes (yes/no/missing), family history of disease (CVD/other diseases/missing), and the first 10 genetic principal components.

**Supplement Table 6.** Additionally adjusted stains hazard ratio and 95% confidence interval of hypertension by plasma FA exposure.

|  | | Quartiles of plasma FA (% of total fatty acids) | | | | |  | |
| --- | --- | --- | --- | --- | --- | --- | --- | --- |
|  | Quantile 1 | | Quantile 2 | Quantile 3 | Quantile 4 | *P* trend | | HR (95% CI) |
| PUFAs |  | |  |  |  |  | |  |
| Model 1 | 1.00 | | 0.753 (0.732, 0.774) | 0.618 (0.600, 0.637) | 0.541 (0.524, 0.559) | <0.001 | | 0.789 (0.781, 0.797) |
| Model 2 | 1.00 | | 0.865 (0.841, 0.890) | 0.782 (0.758, 0.806) | 0.711 (0.687, 0.735) | <0.001 | | 0.876 (0.866, 0.886) |
| MUFAs |  | |  |  |  |  | |  |
| Model 1 | 1.00 | | 1.178 (1.138, 1.219) | 1.451 (1.404, 1.500) | 1.915 (1.855, 1.978) | <0.001 | | 1.293 (1.279, 1.307) |
| Model 2 | 1.00 | | 1.082 (1.044, 1.120) | 1.203 (1.163, 1.245) | 1.355 (1.309, 1.403) | <0.001 | | 1.127 (1.114, 1.140) |
| SFAs |  | |  |  |  |  | |  |
| Model 1 | 1.00 | | 0.993 (0.962, 1.026) | 1.098 (1.065, 1.133) | 1.340 (1.300, 1.380) | 0.127 | | 1.135 (1.123, 1.147) |
| Model 2 | 1.00 | | 1.020 (0.987, 1.054) | 1.114 (1.079, 1.150) | 1.252 (1.213, 1.291) | 0.387 | | 1.101 (1.089, 1.112) |
| n-6 PUFAs |  | |  |  |  |  | |  |
| Model 1 | 1.00 | | 1.010 (0.981, 1.040) | 1.023 (0.993, 1.054) | 1.002 (0.972, 1.034) | <0.001 | | 1.016 (1.005, 1.027) |
| Model 2 | 1.00 | | 1.005 (0.975, 1.034) | 1.029 (0.998, 1.060) | 1.021 (0.989, 1.054) | <0.001 | | 1.020 (1.008, 1.031) |
| n-3 PUFAs |  | |  |  |  |  | |  |
| Model 1 | 1.00 | | 0.955 (0.926, 0.984) | 0.915 (0.888, 0.944) | 0.833 (0.807, 0.859) | <0.001 | | 0.930 (0.920, 0.941) |
| Model 2 | 1.00 | | 0.955 (0.926, 0.985) | 0.924 (0.896, 0.953) | 0.891 (0.863, 0.920) | <0.001 | | 0.957 (0.946, 0.968) |
| n-3/n-6 Ratio |  | |  |  |  |  | |  |
| Model 1 | 1.00 | | 0.992 (0.961, 1.023) | 0.963 (0.933, 0.993) | 0.916 (0.888, 0.945) | <0.001 | | 0.992 (0.961, 1.023) |
| Model 2 | 1.00 | | 0.980 (0.950, 1.011) | 0.954 (0.924, 0.985) | 0.937 (0.908, 0.968) | 0.004 | | 0.980 (0.950, 1.011) |

HR, hazard ratio. CI, confidence interval.

Model 1, adjusted for age and gender(male/female).

Model 2, model 1 plus race(White/Asian /Black/other/missing), Townsend deprivation index (TDI), healthy diet (unhealthy/healthy/missing), physical activity(no/yes/unknown), body mass index(BMI) (<25 kg/m2, 25 to 29.9 kg/m2, ≥30 kg/m2, missing), alcohol consumption (daily or almost daily/three or four times a week/once or twice a week/one to three times a month/special occasions only/never/missing), smoking status (never/before/current/missing), diabetes (yes/no/missing), family history of disease (CVD/other diseases/missing) and stains(use/nonuse).

* per 1-SD

**Supplement Table 7.** Adjusted hazard ratio and 95% confidence interval of hypertension by plasma FA exposure excluding participants with were any type of cardiovascular disease at baseline in the UK Biobank.

|  | | Quartiles of plasma FA (% of total fatty acids) | | | | |  | |
| --- | --- | --- | --- | --- | --- | --- | --- | --- |
|  | Quantile 1 | | Quantile 2 | Quantile 3 | Quantile 4 | *P* trend | | HR (95% CI)* |
| PUFAs |  | |  |  |  |  | |  |
| Model 1 | 1.00 | | 0.772 (0.734, 0.813) | 0.606 (0.574, 0.640) | 0.527 (0.497, 0.558) | <0.001 | | 0.781 (0.766, 0.795) |
| Model 2 | 1.00 | | 0.876 (0.832, 0.922) | 0.756 (0.715, 0.800) | 0.687 (0.645, 0.731) | <0.001 | | 0.863 (0.845, 0.881) |
| MUFAs |  | |  |  |  |  | |  |
| Model 1 | 1.00 | | 1.158 (1.087, 1.233) | 1.459 (1.373, 1.550) | 1.898 (1.790, 2.013) | <0.001 | | 1.296 (1.271, 1.322) |
| Model 2 | 1.00 | | 1.064 (0.998, 1.134) | 1.204 (1.131, 1.282) | 1.370 (1.286, 1.460) | 0.001 | | 1.142 (1.117, 1.166) |
| SFAs |  | |  |  |  |  | |  |
| Model 1 | 1.00 | | 1.047 (0.987, 1.112) | 1.150 (1.085, 1.219) | 1.417 (1.340, 1.498) | 0.127 | | 1.154 (1.132, 1.176) |
| Model 2 | 1.00 | | 1.072 (1.010, 1.139) | 1.144 (1.078, 1.215) | 1.318 (1.244, 1.397) | 0.657 | | 1.114 (1.092, 1.136) |
| n-6 PUFAs |  | |  |  |  |  | |  |
| Model 1 | 1.00 | | 0.985 (0.933, 1.040) | 1.053 (0.996, 1.113) | 1.029 (0.972, 1.089) | <0.001 | | 1.022 (1.001, 1.043) |
| Model 2 | 1.00 | | 0.957 (0.906, 1.010) | 1.036 (0.980, 1.095) | 1.001 (0.944, 1.061) | <0.001 | | 1.012 (0.991, 1.034) |
| n-3 PUFAs |  | |  |  |  |  | |  |
| Model 1 | 1.00 | | 0.965 (0.913, 1.020) | 0.872 (0.825, 0.923) | 0.818 (0.773, 0.866) | <0.001 | | 0.921 (0.902, 0.940) |
| Model 2 | 1.00 | | 0.982 (0.928, 1.038) | 0.900 (0.849, 0.953) | 0.905 (0.853, 0.960) | <0.001 | | 0.959 (0.939, 0.980) |
| n-3/n-6 Ratio |  | |  |  |  |  | |  |
| Model 1 | 1.00 | | 1.007 (0.953, 1.065) | 0.910 (0.859, 0.963) | 0.899 (0.849, 0.952) | <0.001 | | 0.960 (0.941, 0.980) |
| Model 2 | 1.00 | | 1.018 (0.962, 1.077) | 0.921 (0.869, 0.976) | 0.960 (0.905, 1.018) | 0.127 | | 0.985 (0.965, 1.006) |

HR, hazard ratio. CI, confidence interval.

Model 1, adjusted for age and gender(male/female).

Model 2, model 1 plus race(White/Asian /Black/other/missing), Townsend deprivation index (TDI), healthy diet (unhealthy/healthy/missing), physical activity(no/yes/unknown), body mass index(BMI) (<25 kg/m2, 25 to 29.9 kg/m2, ≥30 kg/m2, missing), alcohol consumption (daily or almost daily/three or four times a week/once or twice a week/one to three times a month/special occasions only/never/missing), smoking status (never/before/current/missing), diabetes (yes/no/missing) and family history of disease (CVD/other diseases/missing). *

* per 1-SD

**Supplement Table 8.** Adjusted hazard ratio and 95% confidence interval of hypertension by plasma FA exposure excluding participants with follow-up time of less than 2 years in the UK Biobank.

|  | | Quartiles of plasma FA (% of total fatty acids) | | | | |  | |
| --- | --- | --- | --- | --- | --- | --- | --- | --- |
|  | Quantile 1 | | Quantile 2 | Quantile 3 | Quantile 4 | *P* trend | | HR (95% CI)* |
| PUFAs |  | |  |  |  |  | |  |
| Model 1 | 1.00 | | 0.754 (0.733, 0.776) | 0.620 (0.601, 0.640) | 0.544 (0.526, 0.562) | <0.001 | | 0.790 (0.782, 0.798) |
| Model 2 | 1.00 | | 0.860 (0.835, 0.886) | 0.777 (0.753, 0.803) | 0.718 (0.693, 0.744) | <0.001 | | 0.876 (0.866, 0.887) |
| MUFAs |  | |  |  |  |  | |  |
| Model 1 | 1.00 | | 1.176 (1.135, 1.219) | 1.448 (1.399, 1.499) | 1.899 (1.837, 1.964) | <0.001 | | 1.289 (1.275, 1.303) |
| Model 2 | 1.00 | | 1.073 (1.034, 1.112) | 1.199 (1.157, 1.242) | 1.372 (1.324, 1.423) | <0.001 | | 1.139 (1.125, 1.152) |
| SFAs |  | |  |  |  |  | |  |
| Model 1 | 1.00 | | 1.003 (0.970, 1.037) | 1.110 (1.075, 1.147) | 1.343 (1.302, 1.385) | <0.001 | | 1.135 (1.123, 1.148) |
| Model 2 | 1.00 | | 1.011 (0.977, 1.046) | 1.093 (1.057, 1.130) | 1.209 (1.170, 1.249) | <0.001 | | 1.087 (1.075, 1.099) |
| n-6 PUFAs |  | |  |  |  |  | |  |
| Model 1 | 1.00 | | 1.000 (0.970, 1.031) | 1.021 (0.989, 1.053) | 1.002 (0.970, 1.035) | 0.003 | | 1.018 (1.006, 1.030) |
| Model 2 | 1.00 | | 0.975 (0.946, 1.006) | 0.979 (0.949, 1.010) | 0.947 (0.916, 0.978) | 0.431 | | 0.995 (0.983, 1.007) |
| n-3 PUFAs |  | |  |  |  |  | |  |
| Model 1 | 1.00 | | 0.949 (0.919, 0.979) | 0.909 (0.880, 0.938) | 0.832 (0.806, 0.860) | <0.001 | | 0.931 (0.920, 0.942) |
| Model 2 | 1.00 | | 0.980 (0.949, 1.012) | 0.977 (0.946, 1.009) | 0.960 (0.929, 0.993) | 0.002 | | 0.982 (0.971, 0.994) |
| n-3/n-6 Ratio |  | |  |  |  |  | |  |
| Model 1 | 1.00 | | 0.988 (0.956, 1.020) | 0.953 (0.923, 0.985) | 0.916 (0.887, 0.946) | 0.000 | | 0.967 (0.956, 0.978) |
| Model 2 | 1.00 | | 1.008 (0.976, 1.041) | 1.003 (0.971, 1.036) | 1.013 (0.980, 1.047) | 0.774 | | 0.998 (0.987, 1.009) |

HR, hazard ratio. CI, confidence interval.

Model 1, adjusted for age and gender(male/female).

Model 2, model 1 plus Adjusted for age and gender(male/female), race(White/Asian /Black/other/missing), Townsend deprivation index (TDI), healthy diet (unhealthy/healthy/missing), physical activity(no/yes/unknown), body mass index(BMI) (<25 kg/m2, 25 to 29.9 kg/m2, ≥30 kg/m2, missing), alcohol consumption (daily or almost daily/three or four times a week/once or twice a week/one to three times a month/special occasions only/never/missing), smoking status (never/before/current/missing), diabetes (yes/no/missing) and family history of disease (CVD/other diseases/missing), and the first 10 genetic principal components.

* per 1-SD

**Supplement Table 9.** Adjusted hazard ratio and 95% confidence interval of hypertension by plasma FA exposure only race White participants in the UK Biobank.

|  | | Quartiles of plasma FA (% of total fatty acids) | | | | |  | |
| --- | --- | --- | --- | --- | --- | --- | --- | --- |
|  | Quantile 1 | | Quantile 2 | Quantile 3 | Quantile 4 | *P* trend | | HR (95% CI) |
| PUFAs |  | |  |  |  |  | |  |
| Model 1 | 1.00 | | 0.757 (0.736, 0.779) | 0.615 (0.596, 0.634) | 0.511 (0.494, 0.528) | <0.001 | | 0.776 (0.768, 0.784) |
| Model 2 | 1.00 | | 0.872 (0.847, 0.897) | 0.785 (0.760, 0.810) | 0.715 (0.691, 0.741) | <0.001 | | 0.878 (0.868, 0.888) |
| MUFAs |  | |  |  |  |  | |  |
| Model 1 | 1.00 | | 1.210 (1.167, 1.254) | 1.495 (1.445, 1.547) | 1.989 (1.924, 2.056) | <0.001 | | 1.308 (1.294, 1.323) |
| Model 2 | 1.00 | | 1.080 (1.042, 1.120) | 1.201 (1.159, 1.244) | 1.374 (1.326, 1.424) | <0.001 | | 1.137 (1.123, 1.151) |
| SFAs |  | |  |  |  |  | |  |
| Model 1 | 1.00 | | 1.030 (0.996, 1.065) | 1.132 (1.095, 1.169) | 1.399 (1.356, 1.442) | <0.001 | | 1.153 (1.140, 1.165) |
| Model 2 | 1.00 | | 1.004 (0.971, 1.038) | 1.069 (1.035, 1.105) | 1.203 (1.166, 1.242) | <0.001 | | 1.084 (1.072, 1.097) |
| n-6 PUFAs |  | |  |  |  |  | |  |
| Model 1 | 1.00 | | 1.015 (0.985, 1.046) | 1.027 (0.996, 1.060) | 1.016 (0.984, 1.049) | 0.451 | | 1.019 (1.007, 1.031) |
| Model 2 | 1.00 | | 0.985 (0.955, 1.015) | 0.977 (0.947, 1.008) | 0.954 (0.923, 0.985) | 0.002 | | 0.994 (0.982, 1.006) |
| n-3 PUFAs |  | |  |  |  |  | |  |
| Model 1 | 1.00 | | 0.948 (0.919, 0.978) | 0.903 (0.875, 0.932) | 0.812 (0.786, 0.838) | <0.001 | | 0.922 (0.911, 0.933) |
| Model 2 | 1.00 | | 0.982 (0.952, 1.013) | 0.978 (0.948, 1.010) | 0.956 (0.925, 0.988) | 0.004 | | 0.982 (0.970, 0.993) |
| n-3/n-6 Ratio |  | |  |  |  |  | |  |
| Model 1 | 1.00 | | 0.982 (0.951, 1.014) | 0.955 (0.925, 0.986) | 0.899 (0.871, 0.928) | <0.001 | | 0.961 (0.950, 0.972) |
| Model 2 | 1.00 | | 1.001 (0.970, 1.034) | 1.008 (0.976, 1.041) | 1.007 (0.974, 1.040) | 0.743 | | 0.999 (0.988, 1.010) |

HR, hazard ratio. CI, confidence interval.

Model 1, adjusted for age and gender(male/female).

Model 2, model 1 plus, Townsend deprivation index (TDI), healthy diet (unhealthy/healthy/missing), physical activity(no/yes/unknown), body mass index(BMI) (<25 kg/m2, 25 to 29.9 kg/m2, ≥30 kg/m2, missing), alcohol consumption (daily or almost daily/three or four times a week/once or twice a week/one to three times a month/special occasions only/never/missing), smoking status (never/before/current/missing), diabetes (yes/no/missing) and family history of disease (CVD/other diseases/missing).

* per 1-SD

**Supplementary Table 10.** RERI and AP for additive interaction between shift work exposure and genetic categories additionally adjusted stains.

|  |  | Intermediate Risk | | High Risk | |
| --- | --- | --- | --- | --- | --- |
|  |  | RERI (95% CI) | AP (95% CI) | RERI (95% CI) | AP (95% CI) |
| PUFAs | Quantile 3 | 0.078 (-0.118, 0.274) | 0.053 (-0.081, 0.187) | 0.263 (0.045, 0.481) | 0.127 (0.023, 0.232) |
|  | Quantile 2 | 0.132 (-0.067, 0.331) | 0.075 (-0.038, 0.188) | 0.070 (-0.152, 0.292) | 0.033 (-0.071, 0.137) |
|  | Quantile 1 | 0.095 (-0.107, 0.297) | 0.049 (-0.055, 0.152) | 0.224 (-0.002, 0.451) | 0.089 (-0.001, 0.178) |
| MUFAs | Quantile 1 | 0.188 (0.008, 0.368) | 0.134 (0.006, 0.262) | 0.111 (-0.098, 0.320) | 0.061 (-0.053, 0.175) |
|  | Quantile 2 | 0.235 (0.056, 0.415) | 0.149 (0.035, 0.263) | 0.111 (-0.096, 0.318) | 0.056 (-0.049, 0.161) |
|  | Quantile 3 | 0.218 (0.033, 0.403) | 0.122 (0.018, 0.225) | 0.185 (-0.027, 0.397) | 0.082 (-0.012, 0.175) |
| SFAs | Quantile 1 | -0.119 (-0.322, 0.084) | -0.079 (-0.215, 0.056) | 0.088 (-0.126, 0.303) | 0.043 (-0.061, 0.147) |
|  | Quantile 2 | 0.032 (-0.170, 0.233) | 0.018 (-0.097, 0.133) | -0.027 (-0.245, 0.191) | -0.013 (-0.120, 0.093) |
|  | Quantile 3 | -0.111 (-0.318, 0.096) | -0.060 (-0.173, 0.052) | 0.095 (-0.125, 0.314) | 0.039 (-0.052, 0.131) |
| n-6 PUFAs | Quantile 3 | 0.059 (-0.119, 0.236) | 0.040 (-0.082, 0.163) | 0.000 (-0.195, 0.196) | 0.000 (-0.107, 0.107) |
|  | Quantile 2 | -0.028 (-0.202, 0.147) | -0.021 (-0.152, 0.110) | -0.095 (-0.288, 0.098) | -0.056 (-0.170, 0.058) |
|  | Quantile 1 | -0.040 (-0.215, 0.136) | -0.028 (-0.154, 0.098) | -0.116 (-0.312, 0.081) | -0.065 (-0.176, 0.046) |
| n-3 PUFAs | Quantile 3 | -0.046 (-0.214, 0.122) | -0.035 (-0.163, 0.093) | -0.025 (-0.210, 0.160) | -0.015 (-0.122, 0.093) |
|  | Quantile 2 | 0.124 (-0.047, 0.295) | 0.083 (-0.030, 0.196) | 0.138 (-0.051, 0.327) | 0.073 (-0.026, 0.171) |
|  | Quantile 1 | 0.103 (-0.071, 0.277) | 0.069 (-0.047, 0.185) | 0.198 (0.008, 0.388) | 0.102 (0.006, 0.198) |
| n-3/n-6 PUFAs | Quantile 3 | -0.030 (-0.192, 0.132) | -0.023 (-0.150, 0.103) | -0.050 (-0.228, 0.129) | -0.031 (-0.141, 0.080) |
|  | Quantile 2 | 0.088 (-0.078, 0.253) | 0.061 (-0.054, 0.177) | 0.161 (-0.020, 0.343) | 0.087 (-0.010, 0.184) |
|  | Quantile 1 | 0.092 (-0.077, 0.261) | 0.065 (-0.054, 0.184) | 0.158 (-0.026, 0.341) | 0.087 (-0.013, 0.187) |

RERI, relative excess risk due to interaction; AP, attributable proportion due to interaction.

Adjusted for age and gender(male/female), race(White/Asian /Black/other/missing), Townsend deprivation index (TDI), healthy diet (unhealthy/healthy/missing), physical activity(Yes/no/unknown), body mass index(BMI) (<25 kg/m2, 25 to 29.9 kg/m2, ≥30 kg/m2, missing), alcohol consumption (daily or almost daily/three or four times a week/once or twice a week/one to three times a month/special occasions only/never/missing), smoking status (never/before/current/missing), diabetes (yes/no/missing), family history of disease (CVD/other diseases/missing), stains(use/nonuse), genotyping batch, and the first 4 genetic principal components.

**Supplementary Table 11.** RERI and AP for additive interaction between shift work exposure and genetic categories excluding participants with were any type of cardiovascular disease at baseline in the UK Biobank.

|  |  | Intermediate Risk | | High Risk | |
| --- | --- | --- | --- | --- | --- |
|  |  | RERI (95% CI) | AP (95% CI) | RERI (95% CI) | AP (95% CI) |
| PUFAs | Quantile 3 | 0.069 (-0.134, 0.272) | 0.046 (-0.090, 0.182) | 0.285 (0.062, 0.508) | 0.135 (0.030, 0.240) |
|  | Quantile 2 | 0.121 (-0.085, 0.327) | 0.068 (-0.048, 0.183) | 0.074 (-0.153, 0.300) | 0.034 (-0.071, 0.139) |
|  | Quantile 1 | 0.119 (-0.088, 0.325) | 0.059 (-0.044, 0.163) | 0.258 (0.027, 0.488) | 0.100 (0.011, 0.189) |
| MUFAs | Quantile 1 | 0.203 (0.020, 0.386) | 0.144 (0.014, 0.274) | 0.117 (-0.096, 0.330) | 0.063 (-0.051, 0.178) |
|  | Quantile 2 | 0.254 (0.071, 0.437) | 0.160 (0.045, 0.275) | 0.095 (-0.116, 0.306) | 0.048 (-0.059, 0.154) |
|  | Quantile 3 | 0.266 (0.078, 0.454) | 0.144 (0.042, 0.247) | 0.189 (-0.028, 0.405) | 0.082 (-0.012, 0.176) |
| SFAs | Quantile 1 | -0.166 (-0.376, 0.045) | -0.111 (-0.251, 0.030) | 0.086 (-0.133, 0.304) | 0.041 (-0.064, 0.146) |
|  | Quantile 2 | -0.002 (-0.209, 0.206) | -0.001 (-0.119, 0.118) | -0.026 (-0.247, 0.196) | -0.013 (-0.120, 0.095) |
|  | Quantile 3 | -0.116 (-0.327, 0.095) | -0.063 (-0.178, 0.051) | 0.104 (-0.117, 0.325) | 0.043 (-0.049, 0.135) |
| n-6 PUFAs | Quantile 3 | 0.077 (-0.105, 0.259) | 0.052 (-0.071, 0.175) | -0.013 (-0.213, 0.187) | -0.007 (-0.115, 0.102) |
|  | Quantile 2 | -0.002 (-0.180, 0.176) | -0.001 (-0.133, 0.130) | -0.077 (-0.274, 0.121) | -0.044 (-0.157, 0.069) |
|  | Quantile 1 | 0.001 (-0.178, 0.180) | 0.001 (-0.123, 0.124) | -0.078 (-0.278, 0.121) | -0.043 (-0.152, 0.066) |
| n-3 PUFAs | Quantile 3 | -0.065 (-0.238, 0.108) | -0.049 (-0.179, 0.081) | -0.074 (-0.264, 0.116) | -0.043 (-0.154, 0.068) |
|  | Quantile 2 | 0.113 (-0.061, 0.286) | 0.075 (-0.040, 0.190) | 0.110 (-0.081, 0.301) | 0.058 (-0.042, 0.158) |
|  | Quantile 1 | 0.042 (-0.135, 0.219) | 0.029 (-0.093, 0.151) | 0.137 (-0.058, 0.331) | 0.070 (-0.029, 0.170) |
| n-3/n-6 PUFAs | Quantile 3 | -0.043 (-0.209, 0.123) | -0.033 (-0.162, 0.096) | -0.101 (-0.284, 0.082) | -0.062 (-0.176, 0.051) |
|  | Quantile 2 | 0.108 (-0.060, 0.277) | 0.075 (-0.041, 0.191) | 0.156 (-0.028, 0.340) | 0.084 (-0.014, 0.181) |
|  | Quantile 1 | 0.049 (-0.122, 0.220) | 0.036 (-0.089, 0.160) | 0.096 (-0.091, 0.282) | 0.053 (-0.050, 0.156) |

RERI, relative excess risk due to interaction; AP, attributable proportion due to interaction.

Adjusted for age and gender(male/female), race(White/Asian /Black/other/missing), Townsend deprivation index (TDI), healthy diet (unhealthy/healthy/missing), physical activity(Yes/no/unknown), body mass index(BMI) (<25 kg/m2, 25 to 29.9 kg/m2, ≥30 kg/m2, missing), alcohol consumption (daily or almost daily/three or four times a week/once or twice a week/one to three times a month/special occasions only/never/missing), smoking status (never/before/current/missing), diabetes (yes/no/missing), family history of disease (CVD/other diseases/missing), and the first 10 genetic principal components.

**Supplementary Table 12.** RERI and AP for additive interaction between shift work exposure and genetic categories excluding participants with follow-up time of less than 2 years in the UK Biobank.

|  |  | Intermediate Risk | | High Risk | |
| --- | --- | --- | --- | --- | --- |
|  |  | RERI (95% CI) | AP (95% CI) | RERI (95% CI) | AP (95% CI) |
| PUFAs | Quantile 3 | 0.114 (0.007, 0.221) | 0.076 (0.005, 0.148) | 0.111 (-0.008, 0.229) | 0.058 (-0.004, 0.120) |
|  | Quantile 2 | 0.036 (-0.072, 0.144) | 0.023 (-0.045, 0.090) | 0.079 (-0.040, 0.198) | 0.038 (-0.019, 0.095) |
|  | Quantile 1 | 0.094 (-0.015, 0.202) | 0.050 (-0.008, 0.108) | 0.143 (0.024, 0.262) | 0.061 (0.010, 0.111) |
| MUFAs | Quantile 1 | 0.121 (0.017, 0.224) | 0.085 (0.012, 0.158) | 0.092 (-0.024, 0.208) | 0.050 (-0.013, 0.113) |
|  | Quantile 2 | 0.116 (0.013, 0.219) | 0.075 (0.008, 0.141) | 0.098 (-0.017, 0.213) | 0.050 (-0.008, 0.107) |
|  | Quantile 3 | 0.156 (0.051, 0.261) | 0.086 (0.028, 0.145) | 0.177 (0.061, 0.293) | 0.078 (0.027, 0.129) |
| SFAs | Quantile 1 | -0.071 (-0.174, 0.032) | -0.051 (-0.127, 0.024) | 0.017 (-0.095, 0.128) | 0.009 (-0.051, 0.069) |
|  | Quantile 2 | 0.010 (-0.093, 0.113) | 0.007 (-0.060, 0.074) | -0.025 (-0.136, 0.087) | -0.013 (-0.073, 0.046) |
|  | Quantile 3 | -0.096 (-0.200, 0.008) | -0.060 (-0.125, 0.005) | 0.039 (-0.072, 0.151) | 0.019 (-0.034, 0.071) |
| n-6 PUFAs | Quantile 3 | -0.003 (-0.101, 0.096) | -0.002 (-0.073, 0.069) | 0.070 (-0.036, 0.175) | 0.038 (-0.020, 0.096) |
|  | Quantile 2 | -0.026 (-0.123, 0.072) | -0.019 (-0.089, 0.052) | 0.028 (-0.078, 0.134) | 0.015 (-0.043, 0.074) |
|  | Quantile 1 | -0.040 (-0.137, 0.058) | -0.028 (-0.096, 0.040) | -0.027 (-0.133, 0.079) | -0.015 (-0.073, 0.044) |
| n-3 PUFAs | Quantile 3 | 0.037 (-0.055, 0.128) | 0.027 (-0.039, 0.093) | 0.001 (-0.099, 0.101) | 0.001 (-0.057, 0.059) |
|  | Quantile 2 | 0.001 (-0.091, 0.093) | 0.001 (-0.068, 0.070) | 0.081 (-0.019, 0.181) | 0.046 (-0.010, 0.102) |
|  | Quantile 1 | 0.086 (-0.006, 0.179) | 0.061 (-0.004, 0.126) | 0.042 (-0.059, 0.144) | 0.024 (-0.034, 0.082) |
| n-3/n-6 PUFAs | Quantile 3 | 0.047 (-0.040, 0.135) | 0.036 (-0.030, 0.101) | 0.025 (-0.070, 0.120) | 0.015 (-0.042, 0.073) |
|  | Quantile 2 | 0.011 (-0.078, 0.100) | 0.009 (-0.061, 0.078) | 0.102 (0.006, 0.197) | 0.059 (0.004, 0.115) |
|  | Quantile 1 | 0.063 (-0.026, 0.152) | 0.048 (-0.020, 0.116) | 0.042 (-0.055, 0.139) | 0.026 (-0.034, 0.085) |

RERI, relative excess risk due to interaction; AP, attributable proportion due to interaction.

Adjusted for age and gender(male/female), race(White/Asian /Black/other/missing), Townsend deprivation index (TDI), healthy diet (unhealthy/healthy/missing), physical activity(Yes/no/unknown), body mass index(BMI) (<25 kg/m2, 25 to 29.9 kg/m2, ≥30 kg/m2, missing), alcohol consumption (daily or almost daily/three or four times a week/once or twice a week/one to three times a month/special occasions only/never/missing), smoking status (never/before/current/missing), diabetes (yes/no/missing), family history of disease (CVD/other diseases/missing), and the first 10 genetic principal components.

**Supplementary Table 13.** RERI and AP for additive interaction between shift work exposure and genetic categories only race White participants in the UK Biobank.

|  |  | Intermediate Risk | | High Risk | |
| --- | --- | --- | --- | --- | --- |
|  |  | RERI (95% CI) | AP (95% CI) | RERI (95% CI) | AP (95% CI) |
| PUFAs | Quantile 3 | 0.128 (0.016, 0.240) | 0.084 (0.010, 0.157) | 0.061 (-0.066, 0.189) | 0.031 (-0.033, 0.096) |
|  | Quantile 2 | 0.059 (-0.054, 0.172) | 0.036 (-0.033, 0.106) | 0.063 (-0.064, 0.190) | 0.029 (-0.030, 0.089) |
|  | Quantile 1 | 0.084 (-0.029, 0.198) | 0.045 (-0.016, 0.105) | 0.074 (-0.053, 0.201) | 0.031 (-0.022, 0.084) |
| MUFAs | Quantile 1 | 0.120 (0.011, 0.229) | 0.082 (0.007, 0.157) | 0.038 (-0.086, 0.163) | 0.020 (-0.046, 0.086) |
|  | Quantile 2 | 0.094 (-0.015, 0.203) | 0.059 (-0.010, 0.129) | 0.056 (-0.068, 0.179) | 0.027 (-0.033, 0.087) |
|  | Quantile 3 | 0.136 (0.026, 0.247) | 0.075 (0.014, 0.135) | 0.110 (-0.015, 0.235) | 0.047 (-0.006, 0.101) |
| SFAs | Quantile 1 | -0.112 (-0.221, -0.003) | -0.081 (-0.160, -0.002) | -0.043 (-0.162, 0.076) | -0.023 (-0.086, 0.040) |
|  | Quantile 2 | 0.019 (-0.089, 0.127) | 0.012 (-0.057, 0.080) | -0.078 (-0.197, 0.041) | -0.041 (-0.103, 0.021) |
|  | Quantile 3 | -0.120 (-0.230, -0.011) | -0.074 (-0.141, -0.007) | -0.017 (-0.136, 0.102) | -0.008 (-0.062, 0.047) |
| n-6 PUFAs | Quantile 3 | -0.017 (-0.118, 0.085) | -0.012 (-0.087, 0.062) | 0.087 (-0.022, 0.195) | 0.047 (-0.012, 0.107) |
|  | Quantile 2 | -0.033 (-0.134, 0.068) | -0.024 (-0.097, 0.049) | 0.013 (-0.096, 0.122) | 0.007 (-0.053, 0.068) |
|  | Quantile 1 | -0.048 (-0.148, 0.053) | -0.034 (-0.105, 0.037) | -0.011 (-0.120, 0.098) | -0.006 (-0.066, 0.054) |
| n-3 PUFAs | Quantile 3 | 0.120 (0.011, 0.229) | 0.082 (0.007, 0.157) | 0.038 (-0.086, 0.163) | 0.020 (-0.046, 0.086) |
|  | Quantile 2 | 0.094 (-0.015, 0.203) | 0.059 (-0.010, 0.129) | 0.056 (-0.068, 0.179) | 0.027 (-0.033, 0.087) |
|  | Quantile 1 | 0.136 (0.026, 0.247) | 0.075 (0.014, 0.135) | 0.110 (-0.015, 0.235) | 0.047 (-0.006, 0.101) |
| n-3/n-6 PUFAs | Quantile 3 | 0.063 (-0.027, 0.154) | 0.047 (-0.020, 0.114) | 0.010 (-0.090, 0.109) | 0.006 (-0.054, 0.065) |
|  | Quantile 2 | 0.007 (-0.085, 0.099) | 0.006 (-0.066, 0.077) | 0.094 (-0.005, 0.194) | 0.054 (-0.003, 0.112) |
|  | Quantile 1 | 0.080 (-0.013, 0.173) | 0.060 (-0.009, 0.128) | 0.033 (-0.069, 0.134) | 0.020 (-0.041, 0.081) |

RERI, relative excess risk due to interaction; AP, attributable proportion due to interaction.

Adjusted for age and gender(male/female), Townsend deprivation index (TDI), healthy diet (unhealthy/healthy/missing), physical activity(Yes/no/unknown), body mass index(BMI) (<25 kg/m2, 25 to 29.9 kg/m2, ≥30 kg/m2, missing), alcohol consumption (daily or almost daily/three or four times a week/once or twice a week/one to three times a month/special occasions only/never/missing), smoking status (never/before/current/missing), diabetes (yes/no/missing), family history of disease (CVD/other diseases/missing), and the first 10 genetic principal components.

1. Said MA, Verweij N, van der Harst P. Associations of Combined Genetic and Lifestyle Risks With Incident Cardiovascular Disease and Diabetes in the UK Biobank Study. JAMA cardiology. 2018 Aug 1;3(8):693-702. eng. Epub 2018/06/30. doi:10.1001/jamacardio.2018.1717. Cited in: Pubmed; PMID 29955826.
